# Terrosamycins A and B, Bioactive Polyether Ionophores from *Streptomyces* sp. RKND004 from Prince Edward Island Sediment

**DOI:** 10.3390/md17060347

**Published:** 2019-06-11

**Authors:** Amanda Sproule, Hebelin Correa, Andreas Decken, Bradley Haltli, Fabrice Berrué, David P. Overy, Russell G. Kerr

**Affiliations:** 1 Department of Chemistry, University of Prince Edward Island, 550 University Avenue, Prince Edward Island, Charlottetown, PE C1A 4P3, Canada; amanda.sproule@canada.ca (A.S.); fabrice.berrue@nrc-cnrc.gc.ca (F.B.); 2 Nautilus Biosciences Croda, 550 University Avenue, Prince Edward Island, Charlottetown, PE C1A 4P3, Canada; hebelin.correa@croda.com (H.C.); bradley.haltli@croda.com (B.H.); 3 Department of Chemistry, University of New Brunswick, 30 Dineen Drive, Fredericton, NB E3B 5A3, Canada; adecken@unb.ca; 4 Department of Biomedical Science, Atlantic Veterinary College, University of Prince Edward Island, 550 University Avenue, Prince Edward Island, Charlottetown, PE C1A 4P3 Canada; 5 Department of Pathology and Microbiology, Atlantic Veterinary College, University of Prince Edward Island, 550 University Avenue, Prince Edward Island, Charlottetown, PE C1A 4P3, Canada; david.overy@canada.ca

**Keywords:** *Streptomyces*, polycyclic polyether, marine sediment, X-ray diffraction, antibiotic, metabolomics

## Abstract

Terrosamycins A (**1**) and B (**2**), two polycyclic polyether natural products, were purified from the fermentation broth of *Streptomyces* sp. RKND004 isolated from Prince Edward Island sediment. The one strain-many compounds (OSMAC) approach coupled with UPLC-HRMS-based metabolomics screening led to the identification of these compounds. The structure of **1** was determined from analysis of NMR, HRMS, and X-ray diffraction data. NMR experiments performed on **2** revealed the presence of two methoxy groups replacing two hydroxy groups in **1**. Like other polyether ionophores, **1** and **2** exhibited excellent antibiotic activity against Gram-positive pathogens. Interestingly, the terrosamycins also exhibited activity against two breast cancer cell lines.

## 1. Introduction

Gram-positive bacteria belonging to the phylum Actinobacteria, notably the *Streptomyces* spp., are well recognized for their prolific production of bioactive natural products with many approved as drugs [1,2,3]. Because the secondary metabolism of bacteria varies with culture conditions, the one strain–many compounds (OSMAC) approach, which involves fermentation of a small number of strains in a relatively large number of culture conditions, is a commonly used technique in the quest for new natural products [4]. The OSMAC approach has proven to be a successful tool to induce production of natural products from silent pathways that are not observed in standard laboratory cultures [5,6]. In an attempt to discover new natural products, we investigated the metabolic profiles of *Streptomyces* sp. RKND004, a strain isolated from Prince Edward Island sediment, cultured in 14 different liquid media. Chemical screening was performed using liquid chromatography-high-resolution mass spectrometry (UHPLC-HRMS) followed by data processing using the open-source software MZmine 2 [7]. Using this method, we identified the induced polycyclic polyether ionophores that we have named terrosamycins A (**1**) and B (**2**) produced by *Streptomyces* sp. RKND004.

With over 120 structures reported, polyether ionophores represent a large group of natural products with a range of bioactivities including antibacterial, antifungal, antiparasitic and antiviral activity [8,9]. Several members of this group, namely lasalocid, monensin, salinomycin, narasin, maduramycin, laidlomycin, and semduramycin, have been developed for commercial use in veterinary medicine as they target bacterial populations in ruminants and coccidial infections in poultry [10]. Although these compounds have been extensively studied and exploited for their antibiotic activity, recent research has revealed that some naturally occurring polyethers possess anticancer properties. In particular, salinomycin has been shown to have the ability to kill cancer stem cells (CSCs) [11]; pre-clinical and pilot clinical trials suggest the possibility for development as a CSC-targeting agent against a broad spectrum of cancers [12]. 

Herein, we report the isolation and structure elucidation of terrosamycins A (**1**) and B (**2**), induced polyether ionophores exhibiting antibiotic activity comparable to commercially used drugs, as well as new and selective cytotoxicity observed in two breast cancer cell lines. Though the structure of **1** has been previously reported in a patent, limited structural and bioactivity data was disclosed and the patent has since expired [13,14]. Furthermore, **2** is a new analogue. The cytotoxicities of both **1** and **2** have not been reported. Our data suggest the terrosamycins are interesting candidates for further development as antibiotic and/or anticancer agents.

## 2. Results and Discussion

### 2.1. Fermentations and Chemical Screening

*Streptomyces* sp. RKND004 was fermented on a small scale in triplicate in 14 different liquid media for five days. The media included ISP2 as well as 13 bacterial fermentation media (BFM1-11, 13 and 14) which are routinely used in our laboratory (Table S1) [15,16,17,18,19,20,21,22,23,24,25,26]. The culture broths, as well as uninoculated negative controls for each media type, were extracted with ethyl acetate and the extracts analyzed by UHPLC-HRMS. Data processing with the open-source software MZmine 2 [7] was carried out in a manner similar to our previously described method [27] with the addition of chromatogram deconvolution, linear normalization and gap filling steps. In the final aligned peak list, variables (mass features with a defined *m*/*z*, retention time and peak area) that were present in negative control samples as well as methanol blanks used in the UHPLC-HRMS analysis were manually removed. A total of 103 variables remained. 

**Figure 1:**
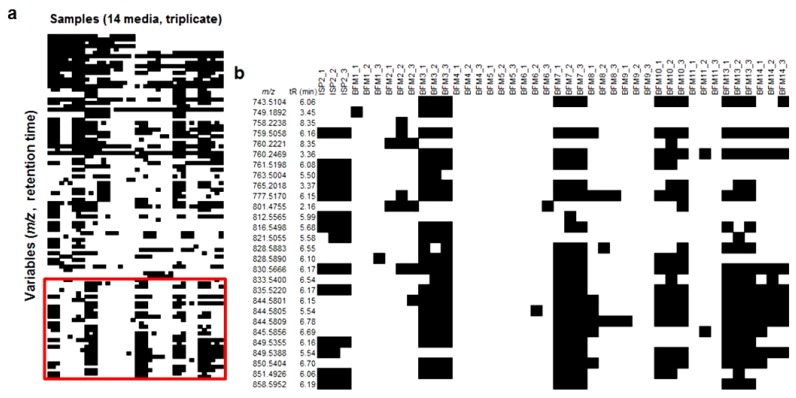
(**a**) Chemical barcode generated for *Streptomyces* sp. RKND004 grown in 14 different media in triplicate and (**b**) an enlarged section of the barcode showing a family of relatively large molecular weight metabolites produced selectively in media ISP2, BFM3, BFM7, BFM10, BFM13 and BFM14. Horizontal rows represent different *m*/*z* and *t*_R_ cells, and vertical columns represent different media (in triplicate).

The resulting processed data set was exported into Microsoft Excel as a two-dimensional data matrix displaying the peak area of each defined variable for each sample. Using conditional formatting in a similar manner to that previously described [27], cells in the spreadsheet containing a peak area greater than the mass detection threshold (set as 1E4) were coloured black, while those with an area below the threshold were coloured white, creating a presence–absence chemical profile (designated as a barcode) for each strain. The barcode was used as a tool to identify induced metabolites that may be selected for further investigation. A number of variables with a similar *m*/*z* and retention time were detected above the threshold in the same set of media (Figure 1a). Figure 1b shows an enlarged section of the barcode containing this family of relatively high molecular weight ions (*m*/*z* > 700). As natural products with larger molecular weights have a greater likelihood of being novel, and because our *Streptomyces* strain had not yet been explored for natural product production, this family of compounds was prioritized for further study.

**Figure 2:**
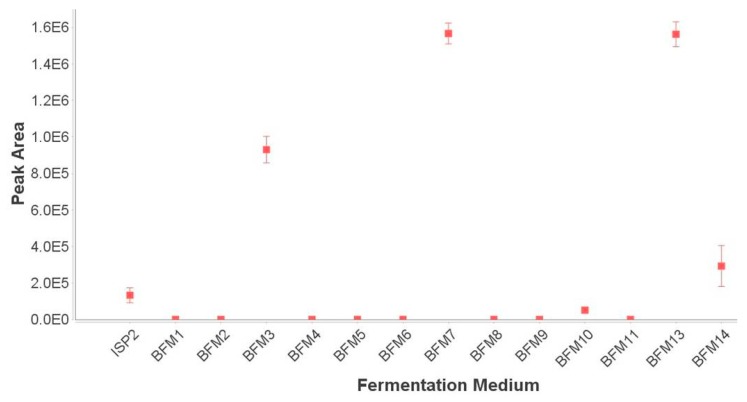
Ion intensity plot displaying the peak area of the [M + Na]^+^ ion *m*/*z* 835.5186 in each different fermentation medium. Data are reported as the average ± SE (*n* = 3).

Analysis of this chemical barcode led to the identification of a broad peak in the UHPLC-HRMS chromatograms for extracts of this strain grown only in ISP2, BFM3, BFM7, BFM10, BFM13 and BFM14. The most abundant compound corresponded to two adduct ions observed in the raw data: *m*/*z* 830.5668 [M + NH_4_]^+^, *t*_R_ 6.20 min and *m*/*z* 835.5186 [M + Na]^+^, *t*_R_ 6.20 min. The [M − H]^−^ ion (*m*/*z* 811.5205, *t*_R_ 6.20 min) was observed when the sample was analyzed in negative mode. A query of AntiBase [28] returned only one match to a compound named N664-30, a polyether ionophore compound reported only once in the literature in a patent containing minimal data [13,14]. An ion intensity plot monitoring the intensity of the [M + Na]^+^ ion was generated using MZmine 2 [7] to better visualize the induction effect (Figure 2) of the culture media. To confirm that the [M + Na]^+^ ion was not excluded from the dataset during data processing by being present below the mass detection threshold, extracted ion chromatograms (XICs) monitoring this ion in the raw data were constructed (Figure S1). The XICs showed that the compound was not produced in BFM1, BFM2, BFM5, BFM6, BFM9 or BFM11, and was present in only trace amounts in BFM4 and BFM8 samples.

### 2.2. Purification and Structure Elucidation

The producing bacterium *Streptomyces* sp. RKND004 was fermented in a total of 9 L of BFM3. The cultures were extracted with ethyl acetate and the resulting deep-red coloured extracts were combined and fractionated by automated flash chromatography using a C_18_ stationary phase. Subsequent fractionation via automated flash chromatography with a silica stationary phase afforded 7.3 mg of **1** and 85.8 mg of **2**, designated as terrosamycin A and terrosamycin B, respectively.

Preliminary NMR data acquired on impure samples during the purification process were consistent with a polyether structure, suggesting that the terrosamycins may belong to this family of natural products. Polyether natural products contain a carboxylic acid moiety and many stereocentres and thus structure elucidation is most often achieved through conversion to a salt, crystallization and single crystal X-ray analysis [8,9]. Salts of **1** and **2** were prepared using sodium hydroxide (containing trace amounts of potassium hydroxide) and subjected to a number of different crystallization conditions in an attempt to obtain crystals suitable for diffraction experiments. Crystals of the terrosamycin A salt were obtained using a slow evaporation method with acetone and deionized water at 4 °C and X-ray analysis and structure refinement was performed (Tables S1–S3).

**Figure 3:**
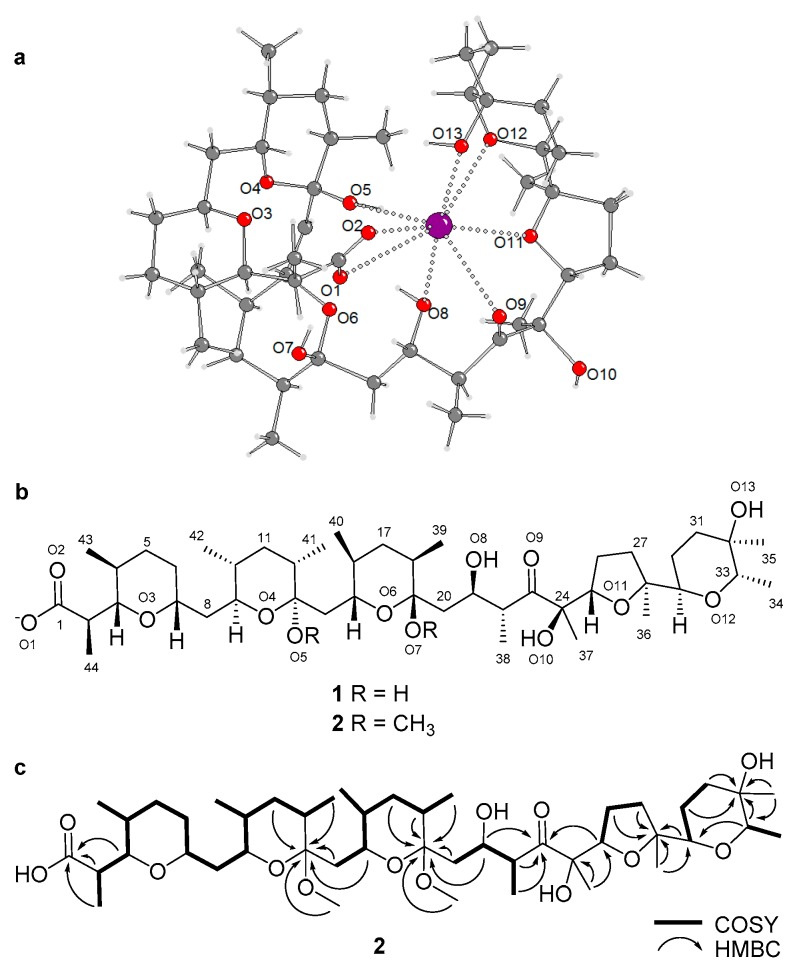
(**a**) Crystal structure of terrosamycin A (**1**) showing one of the independent molecules in the asymmetric unit. Dotted bonds depict the coordination bonds between oxygen (red) and potassium (purple). (**b**) Molecular structure of the terrosamycin A (**1**) and B (**2**) anions. (**c**) Key COSY and HMBC correlations used to elucidate the structure of terrosamycin B (**2**). Similar correlations were used to confirm the structure of terrosamycin A. The numbering scheme used for the crystal data is consistent with the 2D structure.

The X-ray analysis revealed that terrosamycin A (**1**) is a polyether compound. The unit cell of the crystal contained two independent molecules of **1**. The structure features one five-membered and four six-membered heterocyclic rings containing ether linkages. Figure 3a,b indicate the absolute configuration of **1**. As is common to all compounds in this family, a carboxylic acid functional group is located at one terminus of the molecule. Terrosamycin A (**1**) contains a ketone functionality as well as five hydroxy groups, two of which are bound to carbon atoms of ether groups forming cyclic hemiketals. 

Polyether natural products are known to form complexes with monovalent and divalent metal cations [8,9]. The metals can coordinate to oxygen atoms within the structure causing the polyether to wrap itself around the metal resulting in a pseudocyclic structure. The crystal structure of **1** shows complexation of the polyether with a potassium ion. Figure 3a shows coordination bonds from the metal to the O1, O2, O5, O8, O9, O11, O12 and O13 atoms, indicating eight-fold coordination.

The observed coordination to potassium was surprising given that the salt was prepared using aqueous sodium hydroxide. An investigation into the composition of the sodium hydroxide used to prepare the aqueous solution revealed the presence of trace amounts of the potassium salt (up to a concentration of 0.02%). Evidently, **1** may selectively bind K^+^ despite a large excess of Na^+^. This phenomenon is not uncommon among members of the polyether family. Salinomycin binds both monovalent and divalent metal cations with an affinity order of K^+^ > Na^+^ > Cs^+^ > Sr^2+^ > Ca^2+^ = Mg^2+^ [9]. Ionomycin is selective for divalent cations in the order Ca^2+^ > Mg^2+^, where binding to Sr^2+^ and Ba^2+^ was insignificant [29]. Calcimycin, as the name suggests, has a high affinity for Ca^2+^ but can also form a **2**:**1** complex with Mg^2+^, Ni^2+^ and Zn^2+^ [9]. The size and shape of the cage formed around the metal, as well as the number of ligands available for coordination influences the selectivity of each complex. Cations with an atomic radius that fits the cage will bind easily, while smaller cations must adopt a non-optimal coordination geometry and larger ones must distort the cage in order to fit [9]. Although our observations may suggest a preference for binding potassium over sodium, it is also noted that the wrapping of **1** around the smaller sodium ion could have resulted in unfavourable packing scenarios and thus the sodium salt did not crystallize.

The crystal structure of **2** was not determined, as all attempts at crystallization failed. The presence of a bulky methyl group on O5 may have affected the ability of **2** to bind a metal ion. NMR and MS data were used to elucidate its structure. Both **1** and **2** were isolated as colourless glasses and HRESIMS analysis supported molecular formulae of C_44_H_75_O_13_ (*m*/*z* 811.5205 [M − H]^−^, Δ = 0.4 ppm) and C_46_H_79_O_13_ (*m*/*z* 839.5504 [M − H]^−^, Δ = 2.6 ppm), respectively. The structure of **1** deduced from the crystallography data contained seven degrees of unsaturation due to the carboxylic acid, keto group and five rings. Based on the suggested molecular formula, **2** also had seven degrees of unsaturation. The NMR data for **1** (Table 1) were in agreement with the crystal structure and the NMR data for **2** (Table 1) revealed similar signals and correlations with the exception of two additional resonances—C-45 (δ_C_ 47.7) and C-46 (δ_C_ 50.0)—and their corresponding protons H-45 (δ_H_ 3.14) and H-46 (δ_H_ 3.27) which had chemical shifts characteristic of methoxy groups. Their locations were confirmed by two HMBC correlations—H-45/C-13 and H-46/C-19. The replacement of the hydroxy groups on the cyclic hemiketal functionalities with methoxy groups was consistent with the molecular weight and predicted molecular formula for **2**. Assuming that **2** is derived from the methylation of **1**, we suggest that the configuration of all 20 stereogenic centres in **2** is the same as in **1**. The key correlations used to determine the molecular structures are shown in Figure 3c.

The equilibrium that can occur between these two compounds raises questions as to whether terrosamycin B is a genuine natural product, or simply the result of the addition of methanol to terrosamycin A under acidic aqueous conditions. Though there are many polyether natural products that possess cyclic hemiketal functional groups [8,9], there are few which possess cyclic ketals. Ketal-containing derivatives of the polyether ionophores K-41 [8], X-14868B [30], maduramycin [31] and mutalomycin [32] have all been found to arise from synthetic modifications, whether performed intentionally or found accidentally. CP-70828 and CP-70228 are the only two reported polyether ionophores containing cyclic ketals that were claimed to be natural products [33]. The authors demonstrated both the purification of the compounds directly from fermentation broth and purposeful conversion between the hemiketal and ketal by treatment with methanol. This being said, it is questionable whether CP-70828 and CP70228 are genuine natural products. The use of large quantities of methanol during their purification may have caused the interconversion. To determine whether **2** was produced by *Streptomyces* sp. RKND004 or is an artifact of the extraction/isolation process, the fermentation broth was extracted and analyzed without the use of methanol at any stage. An XIC revealed that **2** was present in the EtOAc extract despite never contacting methanol, which suggests that it is in fact a natural product (Figure S16). An increasing ratio of **2**:**1** during purification suggested that the use of acidic aqueous conditions with methanol caused the equilibrium to shift toward formation of **2** and thus increased the overall yield relative to **1**.

The structure of **1**, including the configurations of all stereogenic centres, is identical to that of the patented polyether ionophore N664-30 [14]. The authors determined the structure by X-ray analysis and tentatively reported the compound with “98% confidence”. No X-ray, NMR or other structural data was included in the patent. The patent reported a specific rotation in CHCl_3_ of −5.5° and, in our hands, compound **1** was found to have a value of −18° in the same solvent. While the authors described possible use of the compound as an antibiotic for large farm animals and domestic pets, and as an anticoccidial agent for chickens, there is no mention of any specific bioactivity except that activity against “certain” Gram-positive and Gram-negative microorganisms was observed. Because the patent leaves out many structural and biological properties, and analogue **2** is a new compound, our lab has named both compounds after the Prince Edward Island soil from which their producing bacterium was isolated. The name terrosamycin refers to the red soil on the island. 

### 2.3. Bioactivity

Polyether natural products have been shown to exhibit a wide range of biological effects including antibacterial, antifungal, antiviral and antiparasitic activities [9,34]. Some members of this highly active family have also been explored for their potential as anticancer, anti-inflammatory and herbicidal agents [34]. Additionally, a number of polyethers have immunoregulatory or cardiovascular effects [34]. Polyether natural products are mostly known for their antibiotic activity against Gram-positive bacteria and it is thought that such activity is due to their ability to act as an ionophore. As mentioned earlier, polyether ionophores coordinate to metal cations and bind via the oxygen atoms [8,9]. The resulting complex is cage-like with the hydrophilic oxygen atoms comprising the inner-sphere. The inner-sphere is surrounded by the hydrocarbon backbone, which renders the entire complex hydrophobic [9]. These lipophilic complexes can easily penetrate the cell membrane of Gram-positive bacteria via three separate mechanisms [10]. Cell death arises as a result of the neutral metal-bound complex transporting cations into the cell which disrupts the Na^+^/K^+^ gradient causing the cell to swell and burst [9]. A handful of polyethers have shown activity towards Gram-negative pathogens [9,34]. However, in general, the lipopolysaccharide outer membrane of Gram-negative bacteria acts as an effective barrier against diffusion of these hydrophobic antibiotics [35].

**Figure 4:**
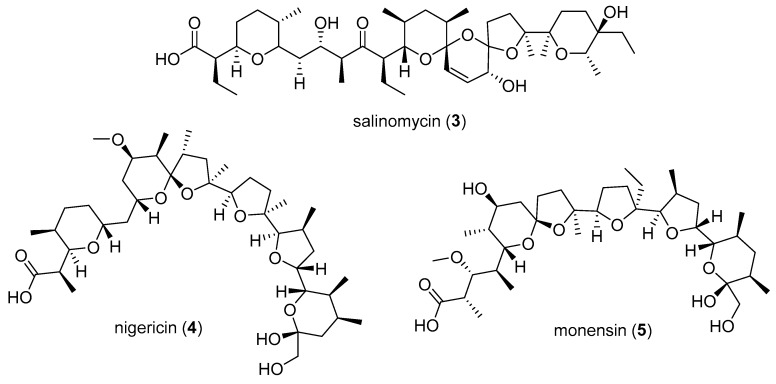
Structures of the polyether natural products salinomycin (**3**), nigericin (**4**) and monensin (**5**).

The antimicrobial activities of **1** and **2** were evaluated against a panel of pathogens. For comparison, three known polyether ionophore standards were selected and tested in parallel. Salinomycin (**3**), nigericin (**4**) and monensin (**5**) (Figure 4) were selected due to the fact that they all exhibit potent antibiotic activity against Gram-positive pathogens and are among the most well studied polyether natural products to date. Furthermore, salinomycin and monensin are currently approved and marketed as antibiotics and anticoccidial agents for cattle and poultry. The compounds were each tested against the Gram-positive pathogens methicillin-resistant Staphyloccocus aureus (MRSA), vancomycin resistant Enterococcus faecium (VRE) and Staphylococcus warneri, Gram-negative pathogens Pseudomonas aeruginosa and Proteus vulgaris, and the yeast Candida albicans. No activity was observed against P. aeruginosa, P. vulgaris or C. albicans at the highest concentration tested (128 μg/mL). Table 2 shows the IC_50_ values for each compound against MRSA, VRE and S. warneri. The results of these antimicrobial assays show that the antibiotic activities of **1** and **2** are on par with commercially used agents.

Despite being mostly known as antibiotic compounds, the anticancer activities of polyether natural products are much less well described with the exception of the 2009 report of selective anticancer activity exhibited by salinomycin (**3**) [11]. Though more work is needed to determine their specific mechanisms of action, many polyethers (including the selected standards) have exhibited potent activity against a broad spectrum of cancer cells, including multi-drug resistant cells and cancer stem cells (CSCs) [10]. Most notably, pilot clinical trials have shown the ability of salinomycin (**3**) to reduce tumor volume and metastasis in four metastatic breast cancer patients as well as a metastatic ovarian cancer patient and a head and neck squamous cell carcinoma patient with no long-term side effects [36]. Monensin (**5**) has been identified as a potent and specific inhibitor of prostate cancer cell growth [37] while nigericin (**4**) has been shown to specifically target CSCs in nasopharyngeal carcinoma and suppress metastasis in colorectal cancer [38,39]. Furthermore, studies indicate that polyethers can increase sensitivity to chemotherapy and possible combinatory therapy is being explored [40].

In light of this information, **1**, **2** and the known polyether standards were tested against a human breast adenocarcinoma cell line (HTB-26) and a human invasive breast ductal carcinoma cell line (MCF-7). The compounds were also tested in a healthy human foreskin fibroblast cell line (BJ) and healthy Cercopithecus aethiops kidney epithelial cells (Vero) as an evaluation of cytotoxicity toward healthy cells. Table 3 displays the IC_50_ values of each compound in each of the cell lines tested. All compounds showed a selective response toward cancer cells when compared to healthy cells. In the HTB-26 cell line, **2** showed the most potent growth inhibition with an IC_50_ value of 6 μM, while **1** and monensin (**5**) had essentially identical activity with values of 10.1 μM and 10 μM, respectively. Similarly, in the MCF-7 cell line, **2** showed the most potent activity while **1** was on par with commercial standards. The difference in activities between **1** and **2** suggests that the methoxy groups of **2** are important for its activity. It is noteworthy to highlight that the activity of the terrosamycins was superior to that of salinomycin (**3**), which has progressed to pilot clinical trials for the treatment of breast cancer, among other cancers [12].

## 3. Materials and Methods

### 3.1. General Experimental Procedures

Optical rotations were measured on a Rudolph Autopol III polarimeter (Rudolph Research Analytical, Hackettstown, NJ, USA) using a 50 mm microcell (1 mL). Infrared spectra were recorded using attenuated total reflectance with samples deposited as a thin film on a Bruker Alpha FT-IR spectrometer (Madison, WI, USA). NMR spectra were obtained on a 600 MHz Bruker Avance III NMR spectrometer equipped with a cryoprobe. Chemical shifts (δ) were referenced to the MeOH-*d*_4_ residual peaks at δ_H_ 3.31 ppm and δ_C_ 49.15 ppm. UHPLC-HRMS data were recorded using Accela Thermo Scientific equipment (ThermoFisher Scientific, Waltham, MA, USA). Fermentation extracts were resuspended in MeOH to a concentration of 500 μg/mL and a 10 μL aliquot was analyzed. A Core Shell 100 Å C_18_ 1.7 um, 50 × 2.1 mm column (Phenomenex, Torrance, CA, USA) and a flow rate of 500 μL/min were used. The solvents used were diH_2_O with 0.1% formic acid (solvent A) and MeCN with 0.1% formic acid (solvent B). Each sample was eluted with a linear solvent gradient from 5% to 100% solvent B over 4.8 min. This was followed by an isocratic elution at 100% solvent B for 3.2 min. Following each run, the system was then returned to the starting conditions over 3 min. Eluent was detected by HRESIMS using an Exactive Orbitrap mass spectrometer (ThermoFisher Scientific, Waltham, MA, USA) monitoring *m*/*z* 190–2000 in positive mode, a Sedex 80 low temperature-evaporative light scattering detector (LT-ELSD) (ThermoFisher Scientific, Waltham, MA, USA), and a photodiode array (PDA) detector (ThermoFisher Scientific, Waltham, MA, USA) monitoring at 200–600 nm. Automated flash chromatography was performed using a Combiflash Rf200 (Teledyne Isco, Lincoln, Nebraska, NE, USA) using C_18_ and silica RediSep columns.

### 3.2. Microbial Material

The strain RKND004 was isolated from a sediment sample composed of fine sand collected in Burnt River, Prince Edward Island in August, 2010 (46.19073° N, 62.48882° W, −141 ft in depth). The strain was fermented in bacterial seed medium (BSM) [18] at 200 rpm and 30 °C for 72 h. Genomic DNA was isolated using a phenol/chloroform/isoamyl alcohol extraction method [41] for use as PCR templates. The 16S rRNA gene was amplified using the 27F and 1525R primers [42] and the *rpoB* gene was amplified using the SRPOF1 and SRPOR1 primers [43]. PCR was performed in total volumes of 25 µL consisting of 12.5 µL EconoTaq PLUS GREEN 2× master mix (Lucigen, Middleton, WI, USA), 1.25 µL DMSO, 7.5 µL diH_2_O, 0.5 µM of the forward and reverse primers and genomic DNA (1.25 µL of a 300 µL dilution). Thermocycler (Eppendorf Mastercycler) conditions were as follows: denaturation at 95 °C for 30 s, 57 °C for 45 s, 72 ° for 1 min, followed by a final extension at 72 °C for 5 min and held at 10 °C for storage. Amplicons were assessed for appropriate size using agarose gel electrophoresis. Sequencing of both 16S rRNA and *rpoB* amplicons was performed by Eurofins MWG Operon (Hunstville, AL, USA). Full-length 16S rRNA gene sequencing (1471 bp) was completed using the primers 16S530R, 16S514F, 16S936R, 16S1525R, 16S1114F and 16S27F [44]. Sequencing of the *rpoB* gene (253 bp) was completed using the primers SRPOF1 and SRPOR1 [43]. Sequences were analyzed and edited using the software program Geneious (Auckland, New Zealand) and compared to existing sequences within the NCBI database using the Basic Local Alignment Search Tool (BLAST) [45]. The full 16S rRNA sequence (GenBank accession number MH420447) was 99% similar to *Streptomyces yanglinensis* 1307 and *Streptomyces paucisporeus* 1412. The *rpoB* sequence (GenBank accession number MH426844) showed 96% similarity to one *Kitasatospora* and 26 *Streptomyces* strains. The strain was therefore labeled as *Streptomyces* sp. RKND004.

### 3.3. Small-Scale Fermentations and Chemical Screening

A two-stage seed protocol was used to generate fermentation inocula. *Streptomyces* sp. RKND004 cells were transferred from an agar plate to 10 mL of BSM [18] (10 g/L dextrose, 4 g/L yeast extract, 0.4 g/L nutrient agar, 15 g/L soluble starch, 1 g/L calcium carbonate, 4 g/L N-Z Amine A) in a 25 × 150 mm culture tube with 4–6 glass beads and incubated at 30 °C with shaking at 200 rpm for 72 h. One milliliter of the first-stage seed culture was used to inoculate the second 10 mL seed culture which was fermented under the same conditions for 48 h. Triplicate tubes of each production medium (10 mL) with 4–6 glass beads were inoculated with 300 μL of the second-stage seed culture and incubated under the same conditions for five days. Uninoculated media blanks were included as negative controls and were subjected to the same incubation conditions. Production media recipes are listed in Table S1. Small-scale fermentations and controls were extracted twice with 10 mL EtOAc with vigorous shaking (200 rpm) at room temperature for 1 h. The organic layers from each extraction were combined and the solvent was removed in vacuo. The extracts were dissolved in MeOH to a concentration of 500 μg/mL and 10 μL aliquots were analyzed by UHPLC-HRMS (ThermoFisher Scientific, Waltham, MA, USA). UHPLC-HRMS data were processed using MZmine 2 [7] as previously described [27] with a number of additions. Briefly, mass detection was performed using the exact mass detector function with an intensity threshold set at 1E4. This was followed by chromatogram building, chromatogram deconvolution, deisotoping, linear normalization, alignment and gap filling steps. For alignment, *m*/*z* tolerance was defined at 0.005 and *t*_R_ tolerance was defined at 0.01 min. The processed peak list was exported as a .CSV file and variables present in the negative controls and MeOH blanks were manually deleted from the peak list. Chemical barcodes were created using conditional formatting in Microsoft Excel, as described previously [27].

### 3.4. Large-Scale Fermentations of Streptomyces sp. RKND004, Extraction and Isolation

Several seed cultures for large-scale fermentations were prepared as described above. *Streptomyces* sp. RKND004 was fermented in a total volume of 9 L (9 × 1 L in Fernbach flasks) of BFM3. Fermentations were inoculated with second-stage seed cultures (2.9% *v*/*v*) and incubated at 30 °C with shaking at 200 rpm for five days. Each culture flask was extracted twice with 400 mL EtOAc overnight at room temperature and the organic layers were combined and evaporated to dryness in vacuo. The resulting extract was fractionated by automated flash chromatography using a Teledyne Isco Combiflash Rf system with a 43 g C_18_ column (High Performance GOLD, RediSep Rf) (Teledyne Isco, Lincoln, NE, USA) and eluted with a flow rate of 20 mL/min. A linear gradient from 1:9 MeOH:diH_2_O to 100% MeOH over 24 min followed by 100% MeOH for 10 min was used and eluent was detected by UV at 214 and 254 nm. Fractions containing 1 and 2 were eluted at 32.5–34.0 min and were combined and further separated via automated flash chromatography with a 12 g silica column (High Performance GOLD, RediSep Rf). The mixture was eluted at 30 mL/min with a gradient of 2:8 acetone:hexanes to 100% acetone over 18 min followed by 100% acetone for 20 min. The eluent was detected by UV at 214 and 254 nm. Terrosamycins A (**1**) and B (**2**) were eluted at 3.0 min and 4.0 min, respectively.

Terrosamycin A (**1**): colourless glass; ${\left[\alpha \right]}_{D}^{28}$ −77 (*c* 0.25, MeOH) and ${\left[\alpha \right]}_{D}^{25}$ −18.2 (*c* 0.00163, CHCl_3_); IR *ν*_max_ 3331, 2968, 2924, 1714, 1577, 1456, 1377, 1105, 1087, 1066, 1042, 1001 cm^−1^; ^1^H and ^13^C NMR data, Table 1; (-) HRESIMS *m*/*z* 811.5205 [M − H]^−^ (calcd. for C_44_H_75_O_13_, 811.52132).

Terrosamycin B (**2**): colourless glass; ${\left[\alpha \right]}_{D}^{27}$ −141 (*c* 1.08, MeOH); IR (film) *ν*_max_ 3479, 2962, 2929, 2854, 1710, 1458, 1378, 1189, 1086, 1067, 1053, 1002 cm^−1^; ^1^H and ^13^C NMR data, Table 1; (-) HRESIMS *m*/*z* 839.5504 [M − H]^−^ (calcd. for C_46_H_79_O_13_, 839.55262).

### 3.5. X-ray Crystallography

The sample of terrosamycin A (**1**) used for NMR experiments was evaporated to dryness and then dissolved in 50% aqueous EtOH, converted to a salt by adding NaOH (with trace KOH) dropwise until the pH was higher than 11 and then dried under a stream of air. The sample was resuspended in 0.5 mL acetone and five drops of diH_2_O and crystals were grown using a slow solvent evaporation method at 4 °C over about one month. The mother liquor was removed with a syringe. Single crystals were coated with Paratone-N oil, mounted using a polyimide MicroMount and frozen in the cold nitrogen stream of the goniometer (−100 °C). A hemisphere of data was collected on a Bruker AXS P4/SMART 1000 diffractometer (Bruker AXS, Madison, WI, USA) using ω and ϕ scans with a scan width of 0.3° and 10 s exposure times. The detector distance was 5 cm. The data were reduced (SAINT) [46] and corrected for absorption (SADABS) [47]. The structure was solved by direct methods and refined by full-matrix least squares on F^2^ (SHELXTL) [48]. The acetone molecule was disordered over two positions. All non-hydrogen atoms were refined using anisotropic displacement parameters. Hydrogen atoms were included in calculated positions and refined using a riding model with the exception of hydrogen atoms of potassium bound hydroxides and the water molecule that were found in Fourier difference maps and refined using bond distance restraints. Crystallographic data for 1 has been deposited with the Cambridge Crystallographic Data Centre. Copies of the data can be obtained, free of charge, on application to the Director, CCDC, 12 Union Road, Cambridge CB2 1EZ, UK (fax: +44-(0)1223-336033 or e-mail: deposit@ccdc.cam.ac.uk).

*Crystal Data for terrosamycin A* (**1**): C_44_H_75_KO_13_·½ Me_2_CO·½ H_2_O; fw = 889.19; monoclinic space group P2(1); unit cell dimensions *a* = 12.004(3) Å, *b* = 35.796(10) Å, *c* = 12.020(3) Å, *V* = 5015(2) Å^3^, *α* = 90°, *β* = 103.826(4)°, *γ* = 90°; *μ*(MoK*^α^*) = 0.166 mm^−1^; *Z* = 4; *D_calc_* = 1.178 Mg/m^3^; 34 091 reflections measured (1.74° ≤ 2*θ* ≤ 27.50°), 21746 independent [*R*_int_ = 0.0760], which were used in all calculations. The final *R*_1_ was 0.0781 [*I* > 2*σ*(*I*)] and *wR*_2_ was 0.1906 (all data). CCDC number: 1843234.

### 3.6. Antimicrobial Assays

The antimicrobial activities of **1**–**5** were evaluated using a panel of human pathogens including methicillin-resistant *S. aureus* ATCC 33591 (MRSA), vancomycin resistant *E. faecium* EF379 (VRE), *S. warneri* ATCC 17917, *P. aeruginosa* ATCC 14210, *P. vulgaris* ATCC 12454 and *C. albicans* ATCC 14035. Assays were performed according to Clinical Laboratory Standards Institute testing standards in a 96-well plate and in triplicate [49]. Compounds **1**–**5** were dissolved in sterile 20% DMSO and serially diluted creating a range of twelve concentrations between 128 μg/mL and 0.0625 μg/mL. A Thermo Scientific Varioskan Flash plate reader was used to determine cell growth by measuring the optical density at 600 nm at time zero and after incubation at 37 °C for 22 h. The change in OD_600_ was calculated, percentages of microorganism survival were compared to the vehicle control wells and IC_50_ values were determined using GraphPad Prism 6.0 (GraphPad Software, San Diego, CA, USA). Control antibiotics used were vancomycin for MRSA and *S. warneri*, rifampicin for VRE, gentamycin for *P. aeruginosa*, ciprofloxacin for *P. vulgaris* and nystatin for *C. albicans*.

### 3.7. Cytotoxicity Assays

The cytotoxicities of compounds **1**–**5** were evaluated using two healthy cell lines—human foreskin fibroblast cells (BJ, ATCC CRL-2522) and *Cercopithecus aethiops* kidney epithelial cells (Vero, ATCC CCL-81)—as well as two cancer cell lines—human breast adenocarcinoma cells (ATCC HTB-26) and human invasive breast ductal carcinoma cells (ATCC MCF-7). Each cell line was grown and maintained in 15 mL of Eagle’s Minimal Essential Medium in T75 cm^2^ cell culture flasks with the exception of HTB-26 cells which were grown in Dulbecco’s Modified Eagle’s Medium/Nutrient Mixture F-12 Ham. All growth media were supplemented with 10% fetal bovine serum, 100 μU/mL penicillin and 0.1 mg/mL streptomycin; the growth medium for MCF-7 cells was also supplemented with 0.01 mg/mL human recombinant insulin. All cell cultures were incubated at 37 °C in a humidified atmosphere with 5% CO_2_ and culture media was refreshed every two to three days. The cells were not allowed to exceed 80% confluency. At 80% confluency, the cells were counted and diluted in their respective growth medium without addition of antibiotics. Aliquots of 90 μL were added to a microwell plate at a density of 1 × 10^4^ cells/well for Vero and HTB-26 cells and 5 × 10^3^ cells/well for BJ fibroblast and MCF-7 cells. The plates were incubated at 37 °C with 5% CO_2_ for 24 h to allow cells to adhere to the plates prior to treatment. The vehicle DMSO was used to dissolve 1 and 2 and was present at a final concentration of 1% in each of the wells. A dilution series was prepared for each cell line using the respective growth medium and 10 μL aliquots of each was added to the respective plate in concentrations ranging from 128 μg/mL to 1 μg/mL in triplicate. Positive control wells contained only the growth medium and DMSO, negative control wells contained the growth medium, DMSO and cells and positive treated controls contained the growth medium, cells and a concentration range of either zinc pyrithione (BJ fibroblast), phenoxyethanol (Vero), or doxorubicin (HTB-26 and MCF-7). All plates were incubated at 37 °C with 5% CO_2_ for 24 h (BJ fibroblast, Vero) or 72 h (HTB-26, MCF-7). Following incubation, alamarBlue was added to each well at a concentration of 10% (*v*/*v*). Fluorescence was measured at 560 nm excitation and 590 nm emission using a Thermo Scientific Varioskan Flash plate reader at time zero and 4 h after treatment with alamarBlue. The change in 590 nm emission reading was used to calculate percent cell viability after each treatment relative to the negative controls. The IC_50_ values of compounds **1**–**5** for each cell line were calculated using GraphPad Prism 6.0.

## 4. Conclusions

In summary, the OSMAC approach coupled with use of our UPLC-HRMS-based chemical barcode tool led to the discovery and isolation of two polyether ionophores, one of which was partially described previously. A combination of X-ray analysis and spectroscopic data allowed us to completely describe the structure, including absolute configuration, of terrosamycin A (**1**) while B (**2**) was solely described using NMR spectroscopy. Notably, terrosamycin A (**1**) exhibited a preference to bind potassium over sodium. Both molecules showed antibiotic activity consistent with other polyether ionophores currently used commercially in the livestock industry. A similar application may be suggested for the terrosamycins. Further, cytotoxicity assays demonstrated that terrosamycins were active against two breast cancer cell lines; notably, the terrosamycins were more active than salinomycin (**3**). These data suggest that the terrosamycins are potential new candidates for anticancer drug development.

## Figures and Tables

**Table 1:** ^1^H (600 MHz) and ^13^C (150 MHz) NMR data for **1** and **2** in MeOH-*d*_4_.

No.	Terrosamycin A (1)			Terrosamycin B (2)		
	δ_C_, Type	δ_H_, (*J*, Hz)	HMBC	δ_C_, Type	δ_H_, (*J*, Hz)	HMBC
1	183.6, C			179.0, C		
2	44.5, CH	2.58, dq (4.4, 7.2, 11.0)	1, 3, 44	42.6, CH	2.64, dq (3.1, 6.8, 9.7)	1, 3, 44
3	86.2, CH	3.66, m	1, 2, 4, 5, 43, 44	84.5, CH	3.45, dd (3.0, 9.8)	1, 2, 5, 43, 44
4	33.4, CH	1.51, m	3, 5, 43, 44	33.6, CH	1.45, m	3, 43
5	33.5, CH_2_	1.47, m	3, 4, 44	34.2, CH_2_	1.53, m	4
		1.28, m	3, 7, 43		1.21, m	3, 4, 7
6	35.2, CH_2_	1.86, m	3, 5, 7	34.3, CH_2_	1.80, m	3, 7
		1.28, m	7		1.27, m	5
7	75.5, CH	3.67, m	3, 6, 8, 9	75.3, CH	3.56, appt ^a^ (10.0)	3, 6, 8, 9
8	39.9, CH_2_	1.67, m	7, 9, 10	41.1, CH_2_	1.62, m	7, 9
		1.15, m	6, 7, 10		1.34, m	9
9	71.5, CH	3.65, m	7, 8, 11, 42	73.7, CH	3.32, td (1.7, 3.3)	7, 8, 11, 42
10	37.7, CH	1.29, m	9, 11, 42	36.8, CH	1.28, m	11
11	38.6, CH_2_	1.37, m	10, 12, 13, 41	38.6, CH_2_	1.42, m	13, 9
		1.30, m	9, 10, 12, 13, 41		1.30, m	9, 14, 41
12	40.4, CH	1.49, m	11, 41	37.0, CH	1.98, m	11, 41
13	98.8, C			102.0, C		
14	44.2, CH_2_	1.82, dd (7.9, 14.8)	12, 13, 15, 16	38.4, CH_2_	1.92, m	12, 13, 15, 16
		1.66, m	13, 16		1.83, m	12, 13, 16
15	72.9, CH	4.10, dd (8.2, 10.1)	13, 14, 17, 40	74.2, CH	3.38, m	13, 14, 17, 40
16	36.9, CH	1.40, m	13, 15, 17, 40	37.1, CH	1.48, m	40
17	38.0, CH_2_	1.48, m	15, 16, 18, 19, 39, 40	38.2, CH_2_	1.37, m	18, 39, 40
					1.29, m	18, 19, 39, 40
18	40.2, CH	1.72, m	17, 19, 20, 39	39.5, CH	1.89, m	17, 39
19	101.3, C			102.6, C		
20	43.1, CH_2_	2.08, dd (1.2, 14.1)	18, 19, 21, 22	39.7, CH_2_	2.08, m	18, 19, 21, 22
		1.57, dd (10.5, 3.2)	18, 19, 21, 22		1.64, m	18, 19, 21, 22
21	74.8, CH	4.22, appt ^a^ (10.0)	19, 20, 22, 23, 38	72.3, CH	4.03, appt ^a^ (8.5)	18, 19, 20, 22, 23, 38
22	45.6, CH	3.5, dq (6.7, 9.8, 13.6)	20, 21, 23, 38	47.8, CH	3.20, dq (5.5, 6.8, 13.8)	19, 20, 21, 23, 38
23	220.7, C			218.0, C		
24	79.9, C			80.9, C		
25	84.8, CH	4.47, dd (5.6, 10.4)	23, 24, 26, 27, 37	84.8, CH	4.24, dd (6.0, 8.8)	23, 24, 26, 27, 37
26	25.8, CH_2_	2.05, m	24, 25, 27, 28	26.7, CH_2_	2.01, m	24
		1.86, m	24, 27, 28		1.93, m	24, 27
27	33.7, CH_2_	2.05, m	25, 26, 28, 29, 36	34.9, CH_2_	2.08, m	26, 28, 29, 36
		1.65, m	25, 26, 28, 36		1.63, m	25, 26, 36
28	86.6, C			86.8, C		
29	74.9, CH	3.56, dd (2.3, 11.8)	27, 28, 30, 31, 33, 36	75.0, CH	3.40, m	27, 28, 30, 33, 36
30	22.0, CH_2_	1.90, m	28, 29, 31, 32	22.9, CH_2_	1.67, m	
		1.60, m	28, 29, 31, 32		1.42, m	32
31	31.8, CH_2_	1.76, td (4.5, 13.5)	29, 30, 32, 33, 35	32.5, CH_2_	1.71, m	30
		1.66, m	29, 30, 32, 33, 35		1.60, m	32, 33
32	70.3, C			70.6, C		
33	79.2, CH	4.08, q (6.5, 13.7)	29, 31, 32, 34, 35	79.0, CH	3.72, q (6.6, 13.5)	29, 34, 35
34	16.1, CH_3_	1.23, d (6.9)	32, 33	15.7, CH_3_	1.19, d (7.3)	32, 33
35	27.2, CH_3_	1.11, s	31, 32, 33	26.6, CH_3_	1.03, s	31, 32, 33
36	26.3, CH_3_	1.22, s	27, 28, 29	23.4, CH_3_	1.12, s	27, 28, 29
37	21.1, CH_3_	1.10, s	23, 24, 25	21.6, CH_3_	1.23, s	23, 24, 25
38	15.0, CH_3_	0.99, d (6.4)	21, 22, 23	15.6, CH_3_	1.12, d (6.7)	21, 22, 23
39	17.0, CH_3_	0.91, d (6.9)	17, 18, 19, 20	17.0, CH_3_	0.88, d (6.5)	17, 18, 19, 20
40	18.7, CH_3_	0.84, d (6.4)	14, 15, 16, 17	19.1, CH_3_	0.86, d (6.5)	14, 15, 16, 17
41	17.2, CH_3_	0.87, d (6.7)	10, 11, 12, 13, 14	17.2, CH_3_	0.87, d (6.2)	10, 11, 12, 13, 14
42	18.0, CH_3_	0.78, d (6.2)	9, 10, 11	18.4, CH_3_	0.79, d (5.5)	9, 10, 11
43	17.5, CH_3_	0.87, d (6.7)	3, 4, 5, 6	18.0, CH_3_	0.84, d (6.8)	3, 4, 5, 6
44	10.7, CH_3_	1.08, d (7.2)	1, 2, 3	9.7, CH_3_	1.08, d (7.6)	1, 2, 3
45				47.7, CH_3_	3.14, s	13
46				50.0, CH_3_	3.27, s	19

^a^ appt indicates an apparent triplet.

**Table 2:** IC_50_ values in μM for terrosamycin A (**1**) and B (**2**) and the polyether standards against methicillin-resistant Staphyloccocus aureus (MRSA), vancomycin resistant Enterococcus faecium (VRE) and S. warneri. Data are reported as the average ± SE (*n* = 6).

Compound	MRSA	VRE	*S. warneri*
terrosamycin A (**1**)	0.5 ± 0.2	0.58 ± 0.04	0.9 ± 0.3
terrosamycin B (**2**)	0.34 ± 0.04	0.5 ± 0.1	0.7 ± 0.1
salinomycin (**3**)	0.4 ± 0.1	0.8 ± 0.2	0.9 ± 0.2
monensin (**4**)	1.5 ± 0.1	8 ± 5	2.0 ± 0.2
nigericin (**5**)	0.2 ± 0.1	0.5 ± 0.1	0.3 ± 0.1
vancomycin control	0.8 ± 0.1	-	0.53 ± 0.01
rifampicin control	-	1.1 ± 0.1	-

**Table 3:** IC_50_ values in μM for terrosamycin A (**1**) and B (**2**) and polyether standards against two breast cancer cell lines (HTB-26, MCF-7) and two healthy cell lines (Vero, BJ). Data are reported as the average ± SE (*n* = 3).

Compound	HTB-26	MCF-7	Vero	BJ
terrosamycin A (**1**)	10.1 ± 0.6	6 ± 1	36 ± 6	79 ± 5
terrosamycin B (**2**)	6 ± 1	3.9 ± 0.4	93 ± 4	267 ± 28
salinomycin (**3**)	13 ± 1	10 ± 3	78 ± 22	196 ± 3
monensin (**4**)	10 ± 1	7 ± 2	1E4 ± 1E3	245 ± 11
nigericin (**5**)	13 ± 2	9 ± 3	61 ± 8	221 ± 10
doxorubicin control	4 ± 2	0.8 ± 0.1	-	-

## Supplementary Materials

The following are available online at https://www.mdpi.com/1660-3397/17/6/347/s1, Figure S1: Extracted ion chromatograms monitoring **1** in extracts from cultures in all media, Figure S2: +ESIMS and −ESIMS spectra of **1**, Figure S3: +ESIMS and −ESIMS spectra of **2**, Figures S4–S8: NMR data of **1**, Figure S9: IR spectrum of **1**, Figures S10–S14: NMR data of **2**, Figure S15: IR spectrum of **2**, Figure S16: Extracted ion chromatogram for **2** in a crude extract analyzed without methanol, Table S1: Production media recipes, Tables S2–S7: X-ray crystal data for **1**.
