# Supplementary material for: Terrosamycins A and B, Bioactive Polyether Ionophores from Streptomyces sp. RKND004 from Prince Edward Island Sediment
Source: Mar Drugs. 2019 Jun 11;17(6):347. doi: 10.3390/md17060347 (PMC6627438; doi:10.3390/md17060347)
Supplement: Supplementary file 1 [file marinedrugs-17-00347-s001.pdf]

Supplementary materials

Terrosamycins A and B, Bioactive Polyether Ionophores from *Streptomyces* sp. RKND004 from Prince Edward Island Sediment

Amanda Sproule [1], Hebelin Correa [2], Andreas Decken [3], Bradley Haltli [2,4], Fabrice Berrue [1], David P. Overy [5] and Russell G. Kerr [1,2,4,*]

1 Department of Chemistry, University of Prince Edward Island, 550 University Avenue, Charlottetown, Prince Edward Island, Canada C1A 4P3; amanda.sproule@canada.ca (A.S.); fabrice.berrue@nrc-cnrc.gc.ca (F.B.)
2 Nautilus Biosciences Croda, 550 University Avenue, Charlottetown, Prince Edward Island, Canada C1A 4P3; hebelin.correa@croda.com (H.C.); bradley.haltli@croda.com (B.H.)
3 Department of Chemistry, University of New Brunswick, 30 Dineen Drive, Fredericton, New Brunswick, Canada E3B 5A3; adecken@unb.ca
4 Department of Biomedical Science, Atlantic Veterinary College, University of Prince Edward Island, 550 University Avenue, Charlottetown, Prince Edward Island, Canada C1A 4P3
5 Department of Pathology and Microbiology, Atlantic Veterinary College, University of Prince Edward Island, 550 University Avenue, Charlottetown, Prince Edward Island, Canada C1A 4P3; david.overy@canada.ca
* Correspondence: rkerr@upei.ca; Tel.: +1-902-566-0565

Table S1. Production media recipes

Figure S1. Extracted ion chromatograms monitoring **1** in extracts from cultures in all media

Figure S2. +ESIMS and -ESIMS spectrum of **1**

Figure S3. +ESIMS and –ESIMS spectrum of **2**

Figure S4. ^{1}H NMR spectrum of **1**

Figure S5. ^{13}C NMR spectrum of **1**

Figure S6. COSY spectrum of **1**

Figure S7. HSQC spectrum of **1**

Figure S8. HMBC spectrum of **1**

Figure S9. IR spectrum of **1**

Figure S10. ^{1}H NMR spectrum of **2**

Figure S11. ^{13}C NMR spectrum of **2**

Figure S12. COSY spectrum of **2**

Figure S13. HQSC spectrum of **2**

Figure S14. HMBC spectrum of **2**

Figure S15. IR spectrum of **2**

Figure S16. Extracted ion chromatogram for **2** in a crude extract analyzed without methanol

Table S2. Crystal data and structure refinement for **1**

Table S3. Atomic coordinates (x10^{4}) and equivalent isotropic displacement parameters for **1**

Table S4. Bond lengths [Å] and angles [°] for **1**

Table S5. Anisotropic displacement parameters (Å^{2}x10^{3}) for **1**

Table S6. Hydrogen coordinates (x10^{4}) and isotropic displacement parameters (Å^{2}x10^{3}) for **1**

Table S7. Hydrogen bonds for terrosamycin A (**1**) [Å and °]

Table S1. Production media recipes

Medium	Ingredient	Quantity (g/L Milli-Q diH_2O, unless otherwise stated)	Specified pH (± 0.2)
ISP2 [15]	yeast extract	2	-
	malt extract	5	
	dextrose	2	
BFM1 [16]	dextrin	20	-
	soluble starch	20	
	beef extract	10	
	tryptone	5	
	ammonium sulfate	2	
	calcium carbonate	2	
BFM2 [17]	soluble starch	5	-
	Pharmamedia	5	
BFM3* [18]	magnesium sulfate heptahydrate	0.5	-
	potassium chloride	0.5	
	dipotassium phosphate	3	
	sodium chloride	5	
	nutrient agar	0.4	
	glycerol	12	
	Bacto™ soytone	5	
BFM4 [19]	toasted Nutrisoy flour	12	6.8
	ammonium chloride	1	
	dextrose	12	
	nutrient agar	0.4	
	calcium carbonate	1	
	N-Z Amine A	3	
BFM5 [20]	tryptone	17	7.3
	Bacto soytone	3	
	sodium chloride	5	
	dipotassium phosphate	2.5	
	dextrose	2.5	

BFM6	yeast extract	5	7.3
	tryptone	5	
	iron (II) chloride tetrahydrate	0.04	
	manganese (II) sulfate monohydrate	0.00034	
	magnesium sulfate heptahydrate	5	
	sodium chloride	58.44	
BFM7* [21]	potassium phosphate	1	7.3
	dipotassium phosphate	1	
	magnesium sulfate heptahydrate	0.2	
	iron (III) chloride	0.05	
	calcium chloride dihydrate	0.02	
	sodium chloride	5	
	glucose	2	
	yeast extract	2	
	olive oil	5	
BFM8* [22]	tryptone	5	7.6
	yeast extract	1	
	ferric citrate	0.1	
BFM9 [23]	yeast extract	5	6.8
	casamino acids	0.1	
	glucose	10	
	sucrose	100	
	potassium sulfate	0.25	
	magnesium chloride hexahydrate	10.2	
	MOPS sodium salt	21	
BFM10 [24]	glycerol	10	7.0
	soybean flour	20	
	corn oil	20	
	dipotassium phosphate	1.2	
	MOPS buffer	21	
	manganese (II) chloride heptahydrate	0.01	
	iron (II) sulfate heptahydrate	0.01	
	zinc sulfate heptahydrate	0.01	

BFM11 [25]	soluble starch	10	7.0
	yeast extract	4	
	tryptone	2	
	potassium bromide stock solution (20 g/L)	5 mL	
	iron (II) sulfate heptahydrate stock solution (8 g/L)	5 mL	
BFM13 [26]	yeast extract	4	-
	dextrose	2	
	sodium chloride	5	
	dipotassium phosphate	2.5	
	potassium phosphate	0.5	
BFM14 [27]	ammonium sulfate	0.39	-
	disodium phosphate	5.67	
	potassium phosphate	4.08	
	calcium chloride dihydrate	0.001	
	magnesium sulfate heptahydrate	0.197	
	manganese (II) sulfate heptahydrate	0.002	
	iron (II) sulfate heptahydrate	0.015	
	Pharmamedia	5	

* Ingredients modified from the original recipes.

A

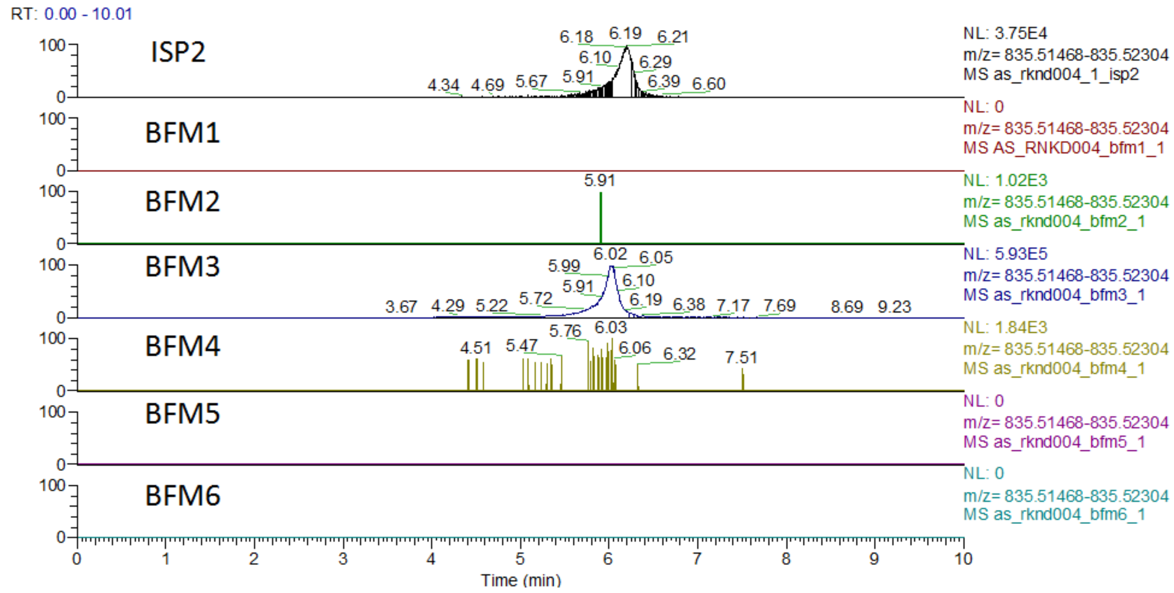

B

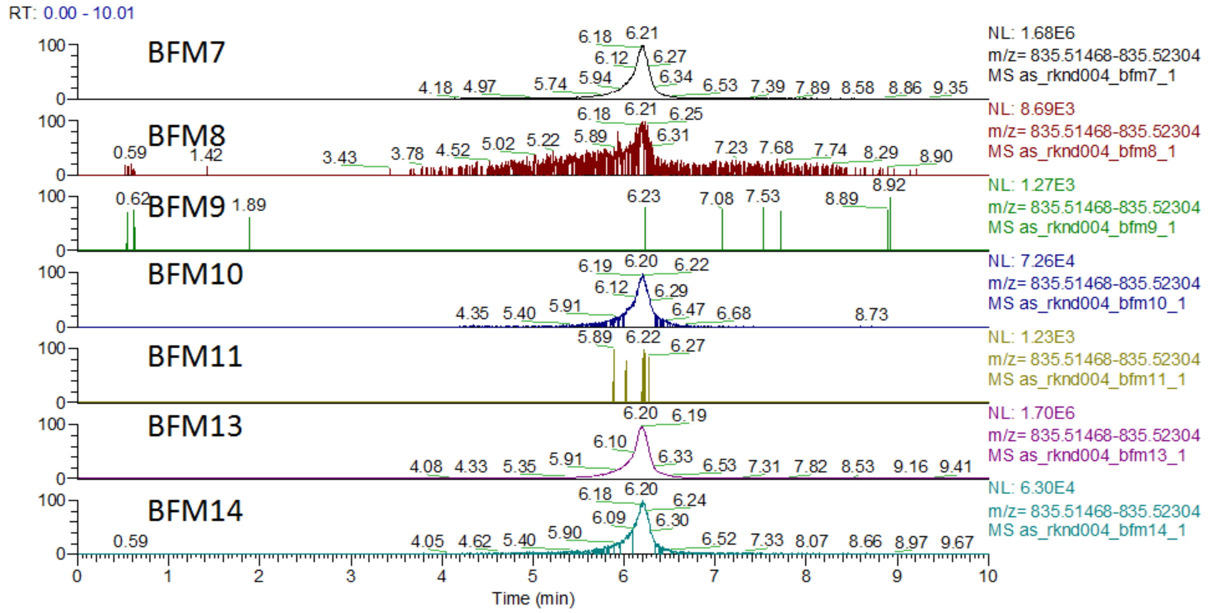

Figure S1. HPLC extracted ion chromatograms (+ESI-HRMS detector) monitoring *m/z* 835.5186 $[M+Na]^+$ in extracts of *Streptomyces* sp. RKND004 grown in **A**. ISP2 and BFM1 – BFM6 and **B**. BFM7 – BFM14. Note the retention times of several metabolites including *m/z* 835.5186 $[M+Na]^+$ detected in the BFM3 sample are slightly shifted compared to those in other media presumably due to matrix effects.

A

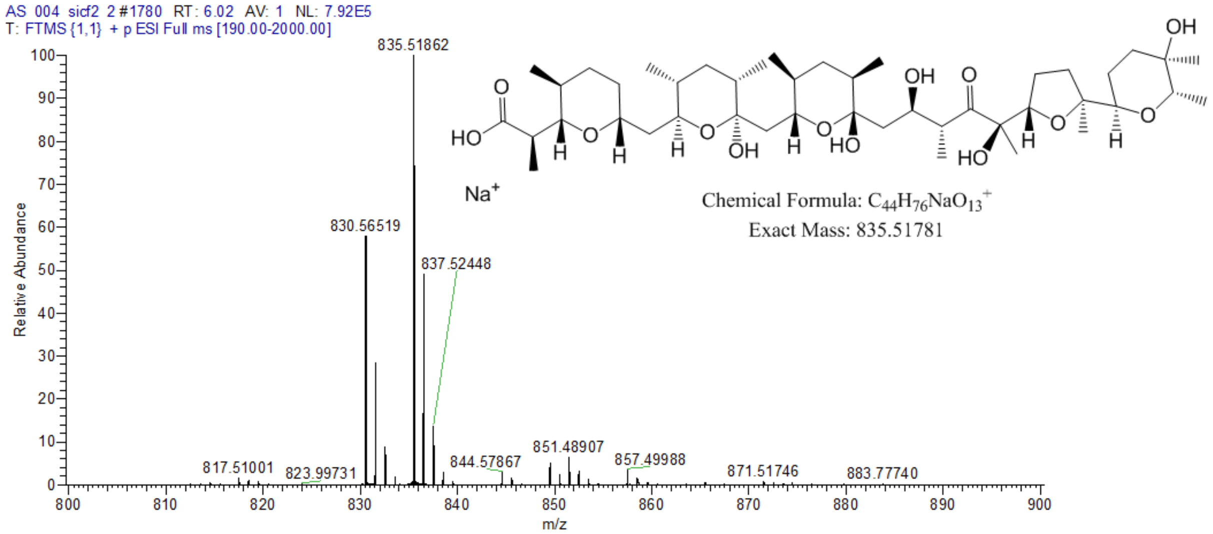

B

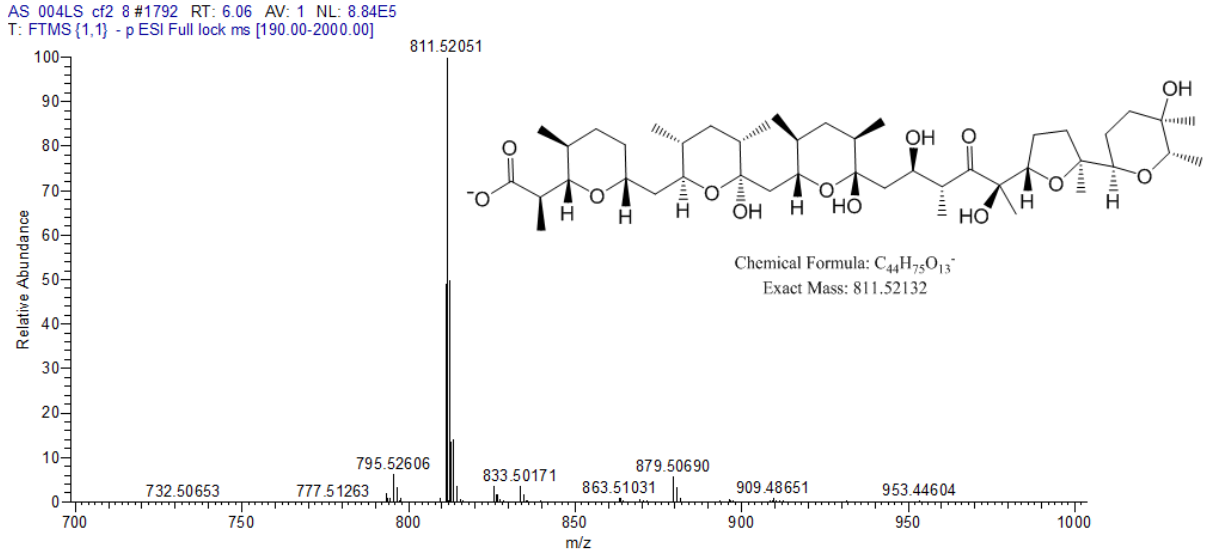

Figure S2. **A**. +ESI-HRMS spectrum of terrosamycin A (**1**).**B**. –ESI-HRMS spectrum of terrosamycin A (**1**).

A

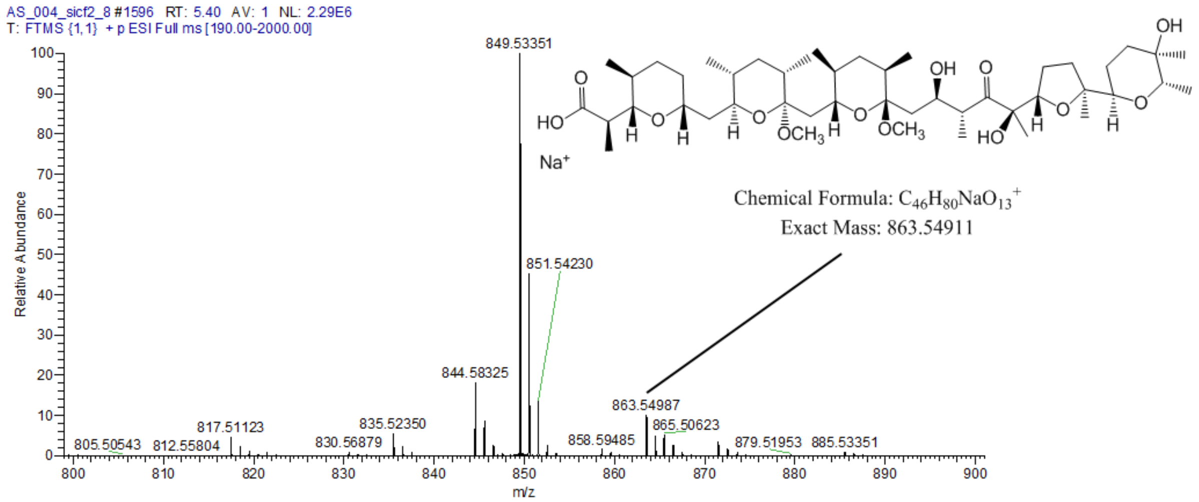

B

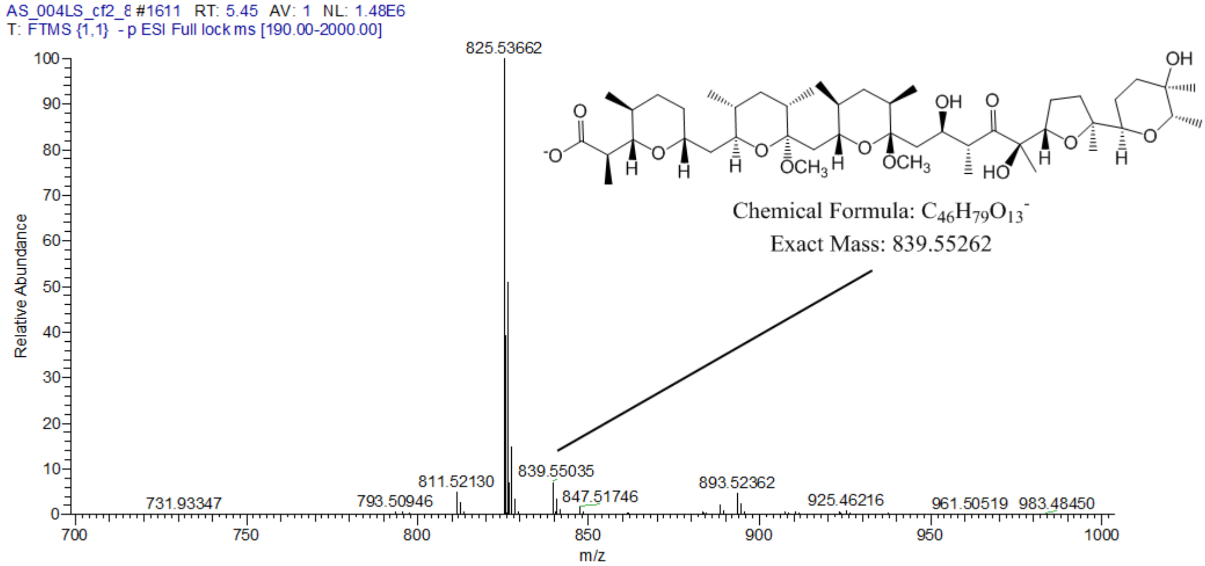

Figure S3. **A**. +ESI-HRMS spectrum of terrosamycin B (**2**). **B**. –ESI-HRMS spectrum of terrosamycin B (**2**).

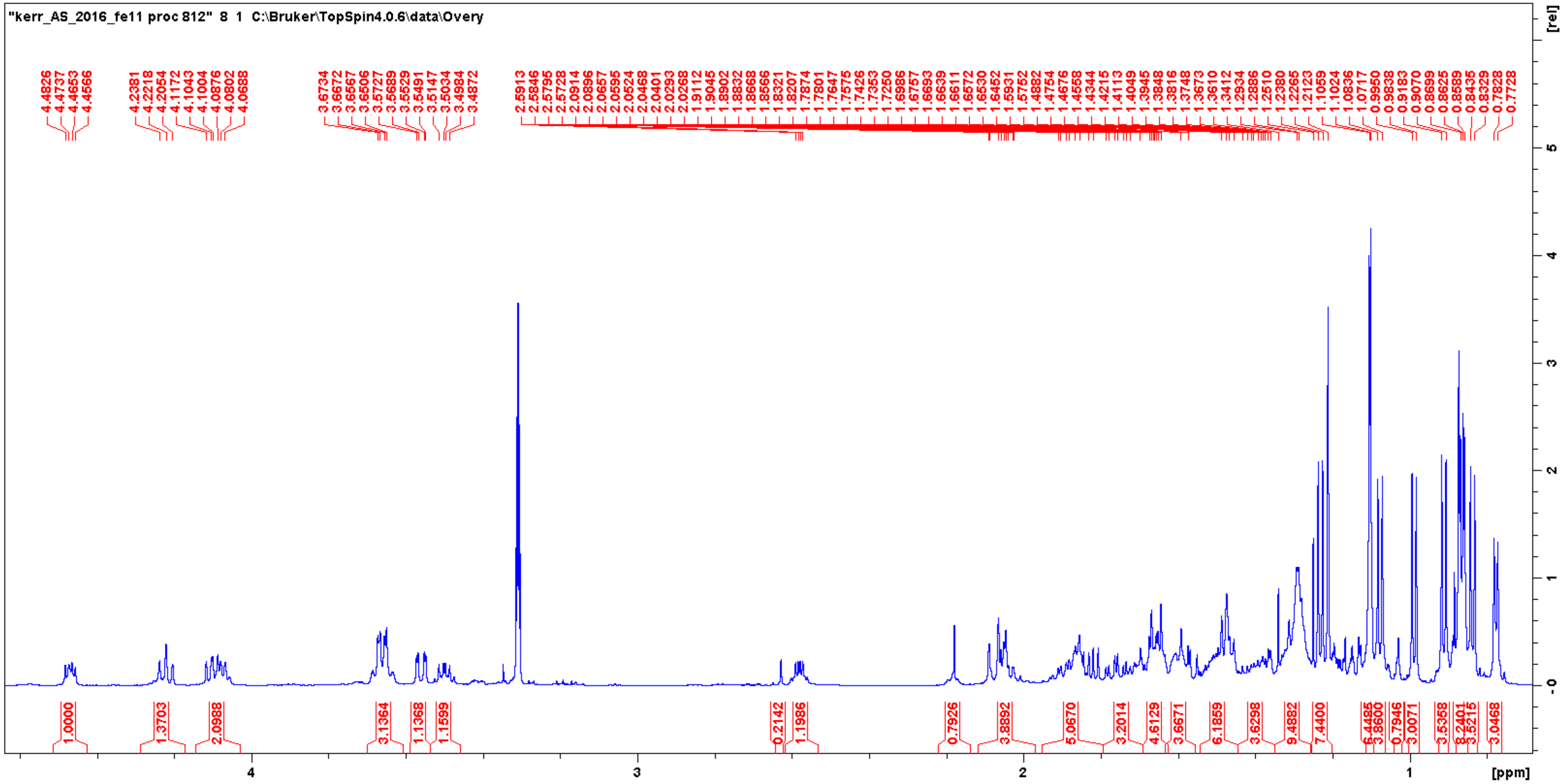

Figure S4. ^{1}H NMR spectrum (600 MHz, MeOD-d4) of terrosamycin A (**1**).

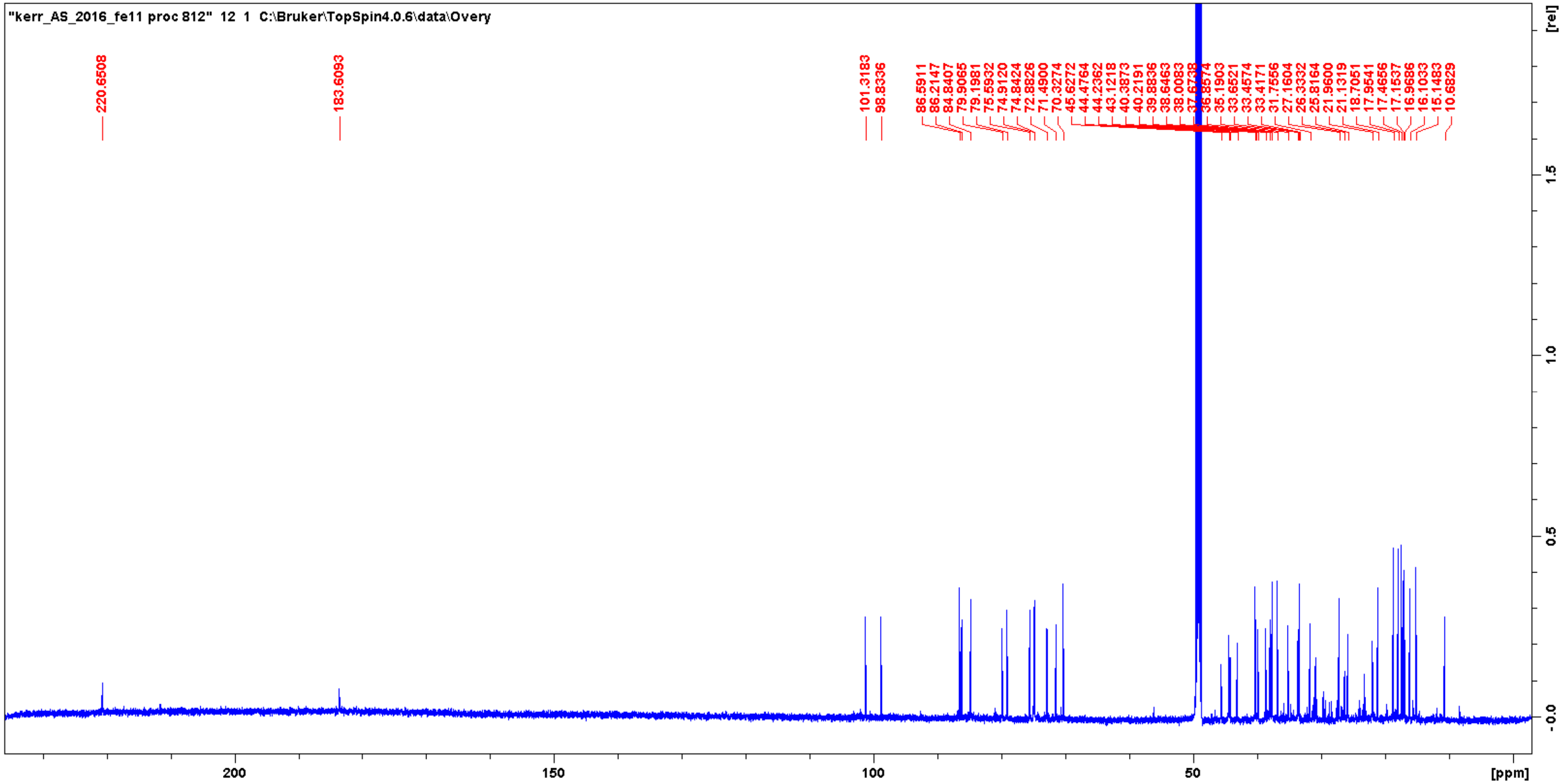

Figure S5. ^{13}C NMR spectrum (150 MHz, MeOD-d$_4$) of terrosamycin A (**1**).

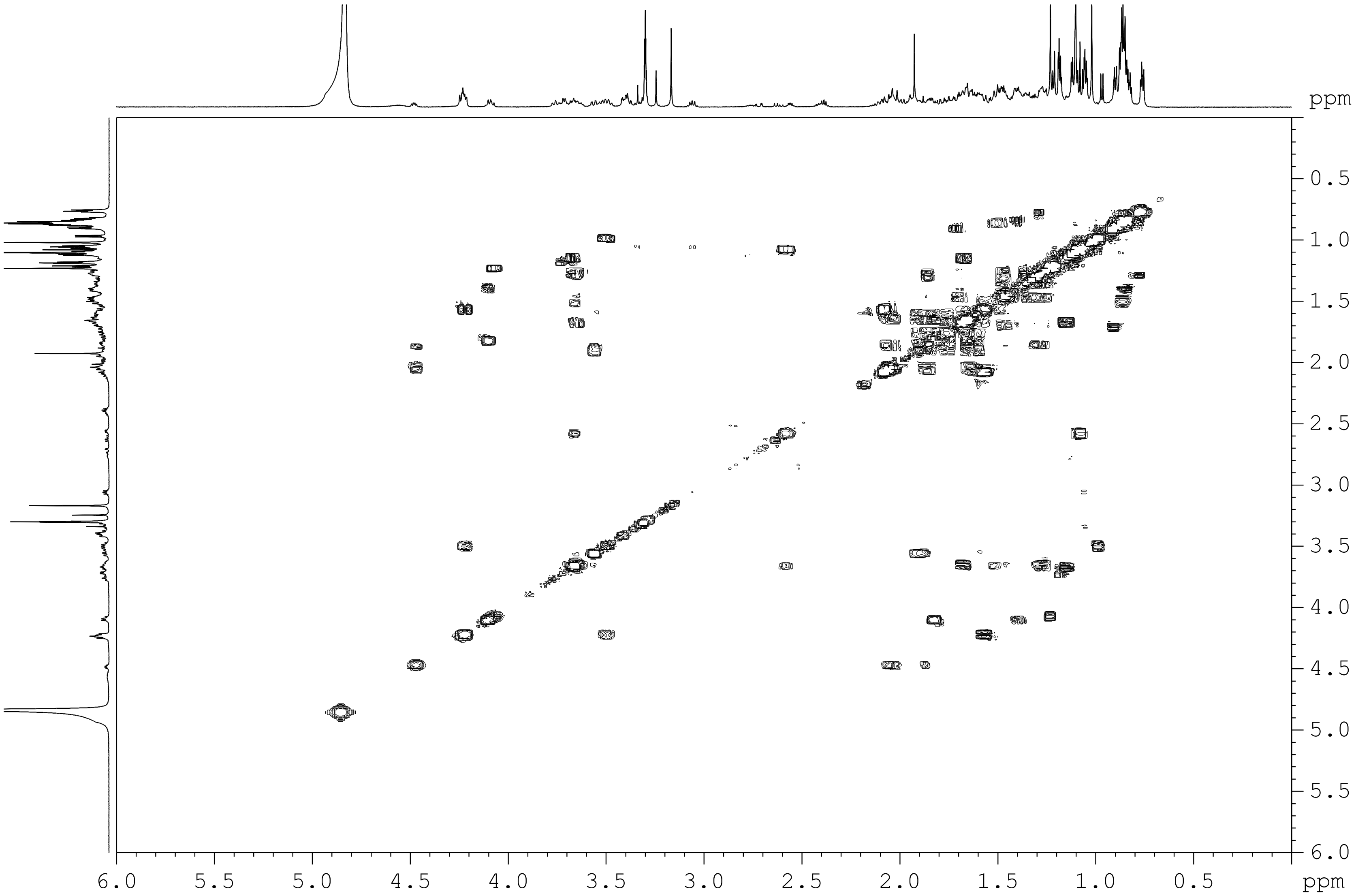

Figure S6. COSY spectrum (600 MHz, MeOD-d4) of terrosamycin A (**1**).

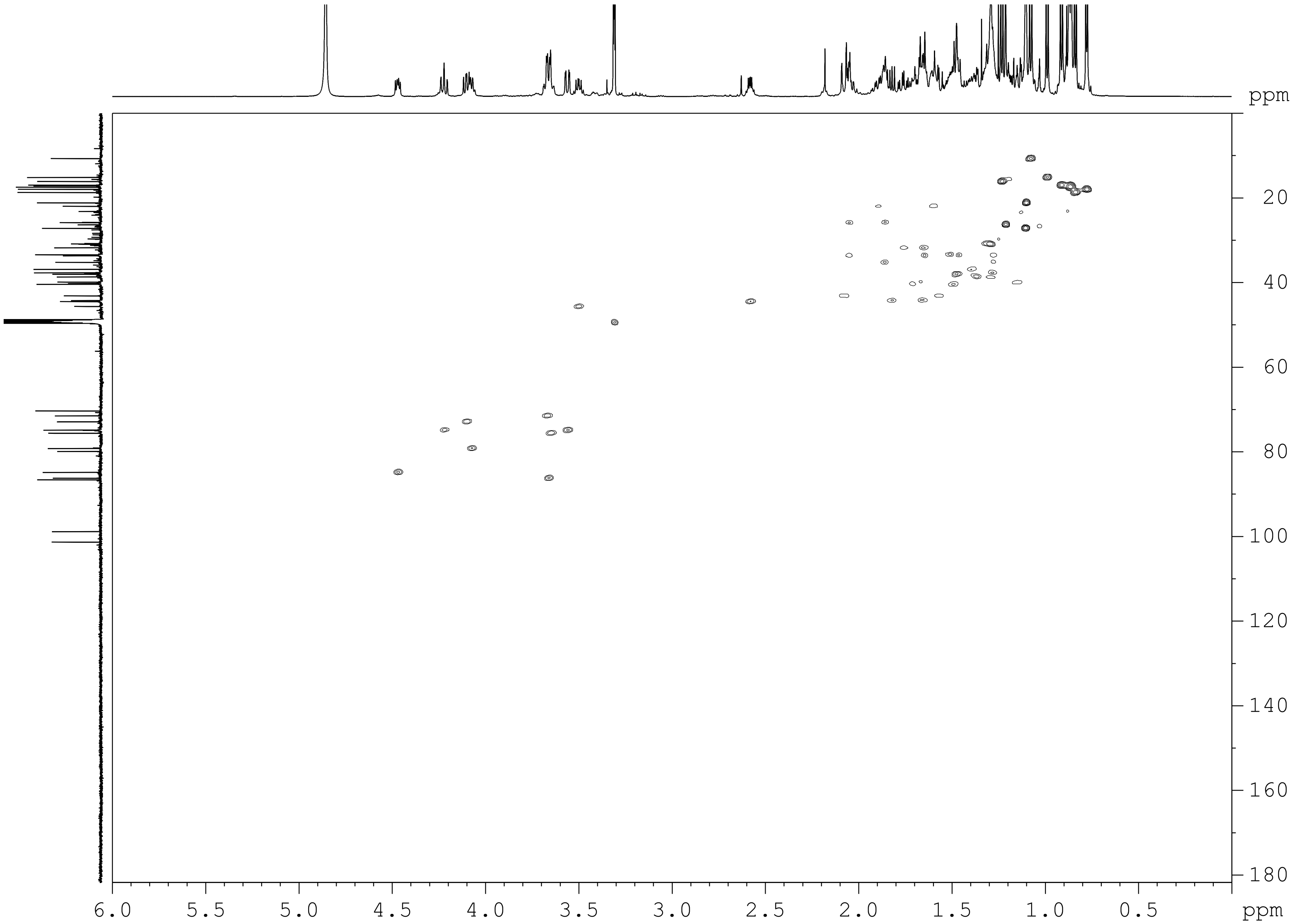

Figure S7. HSQC spectrum (^{1}H 600 MHz, ^{13}C 150 MHz, MeOD-d$_4$) of terrosamycin A (**1**).

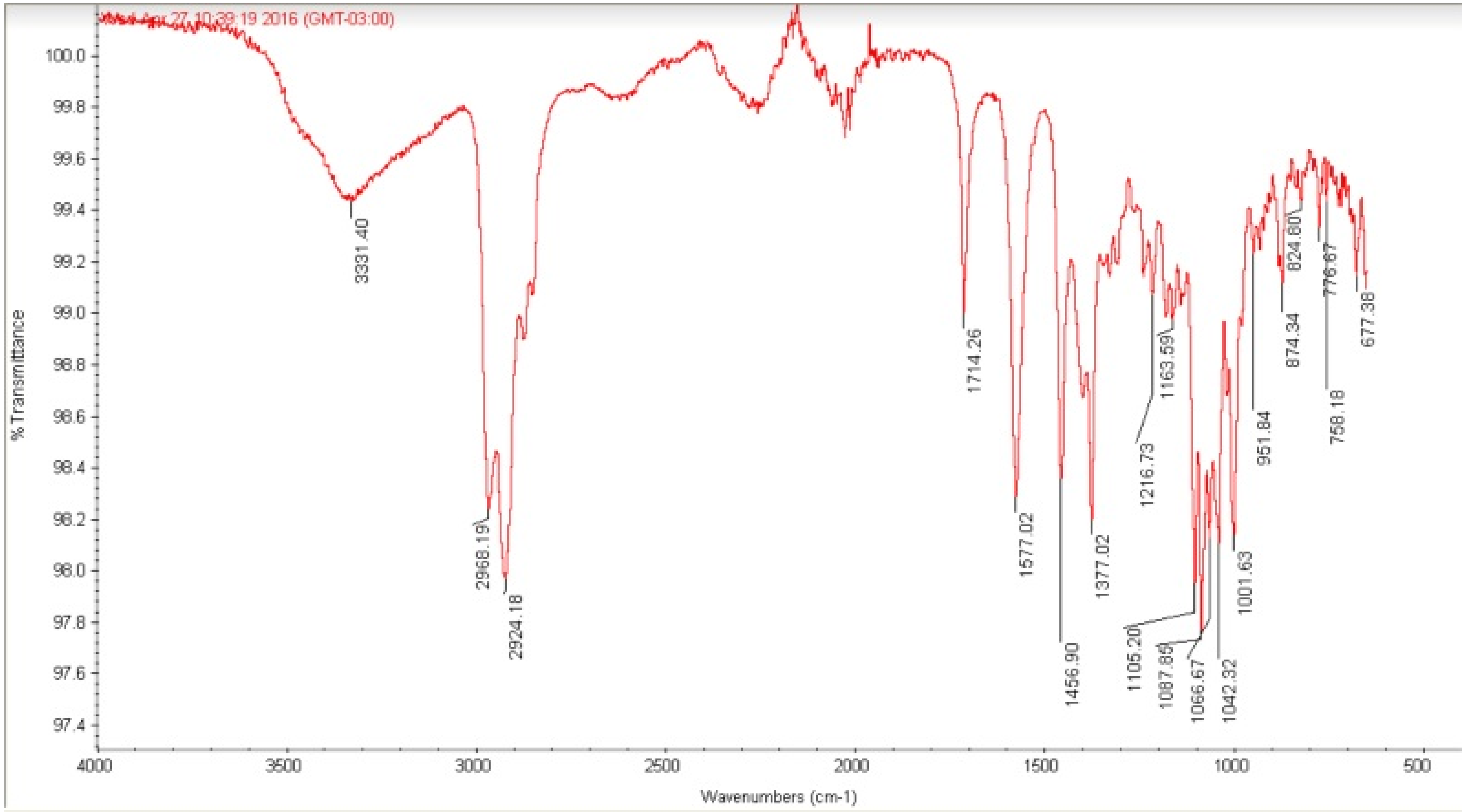

Figure S9. IR spectrum of terrosamycin A (**1**) (MeOH, film).

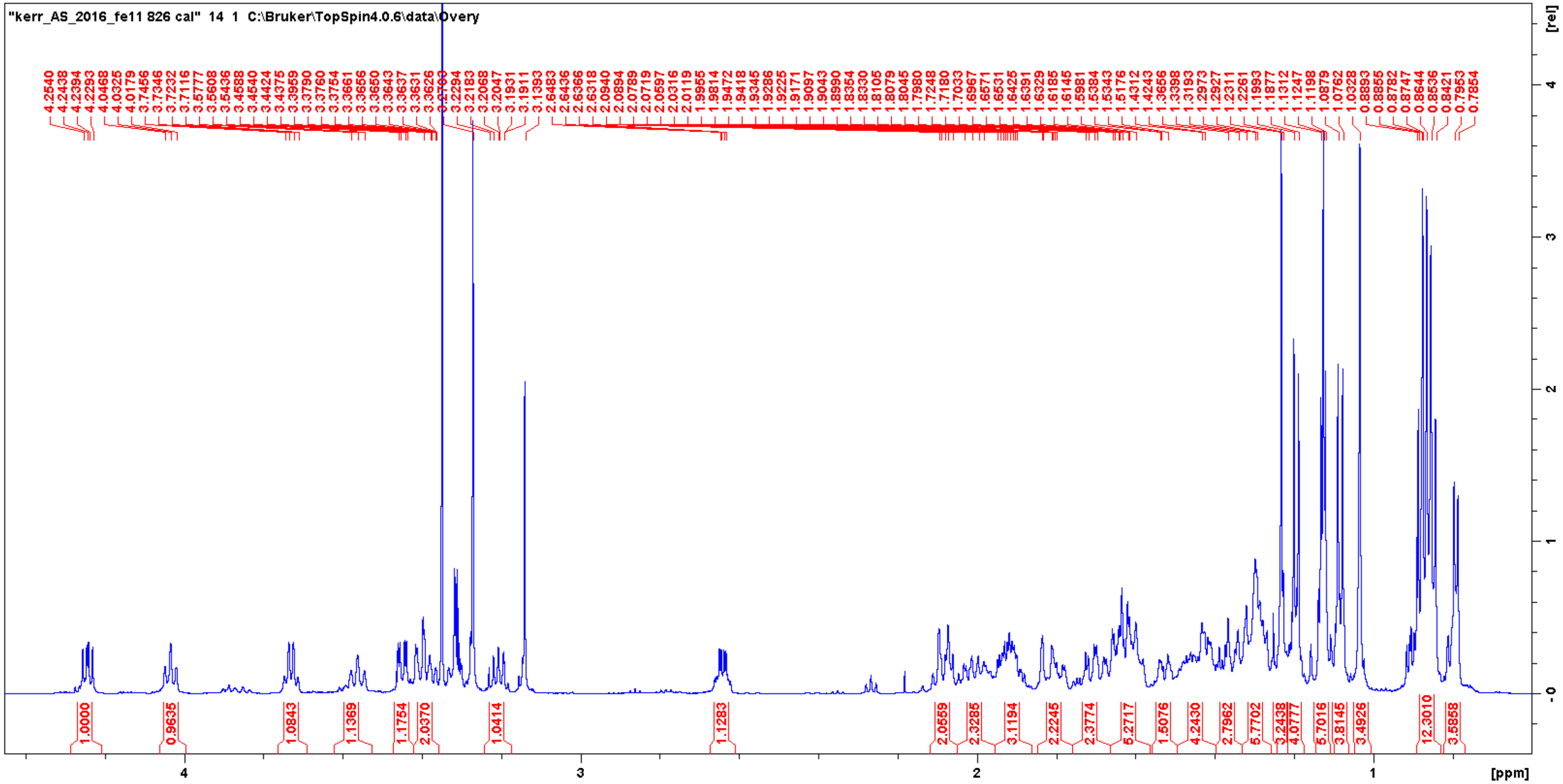

Figure S10. ^{1}H NMR spectrum (600 MHz, MeOD-d_4) of terrosamycin B (**2**).

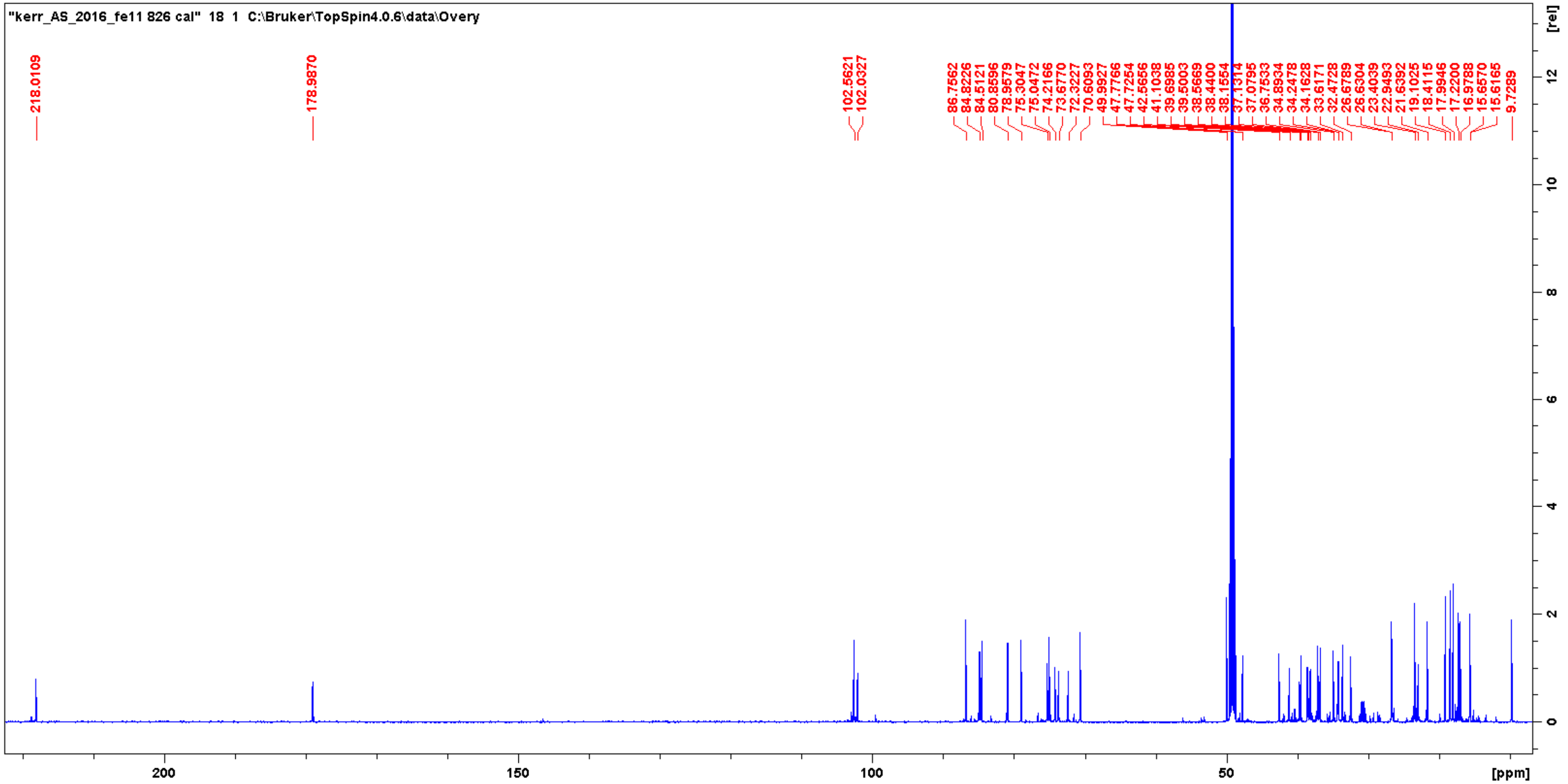

Figure S11. ^{13}C NMR spectrum (150 MHz, MeOD-d$_4$) of terrosamycin B (**2**).

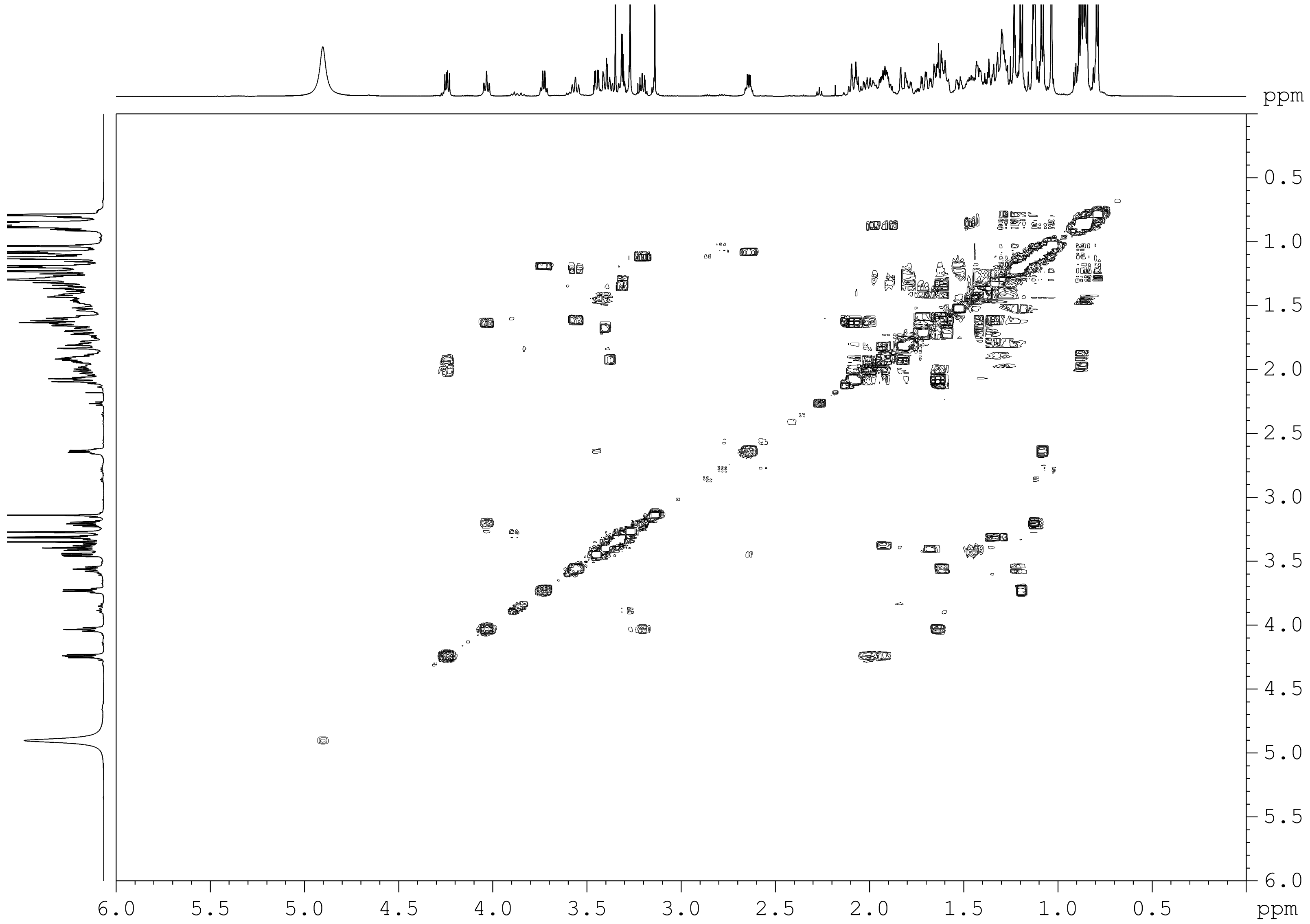

Figure S12. COSY spectrum (600 MHz, MeOD-d4) of terrosamycin B (**2**).

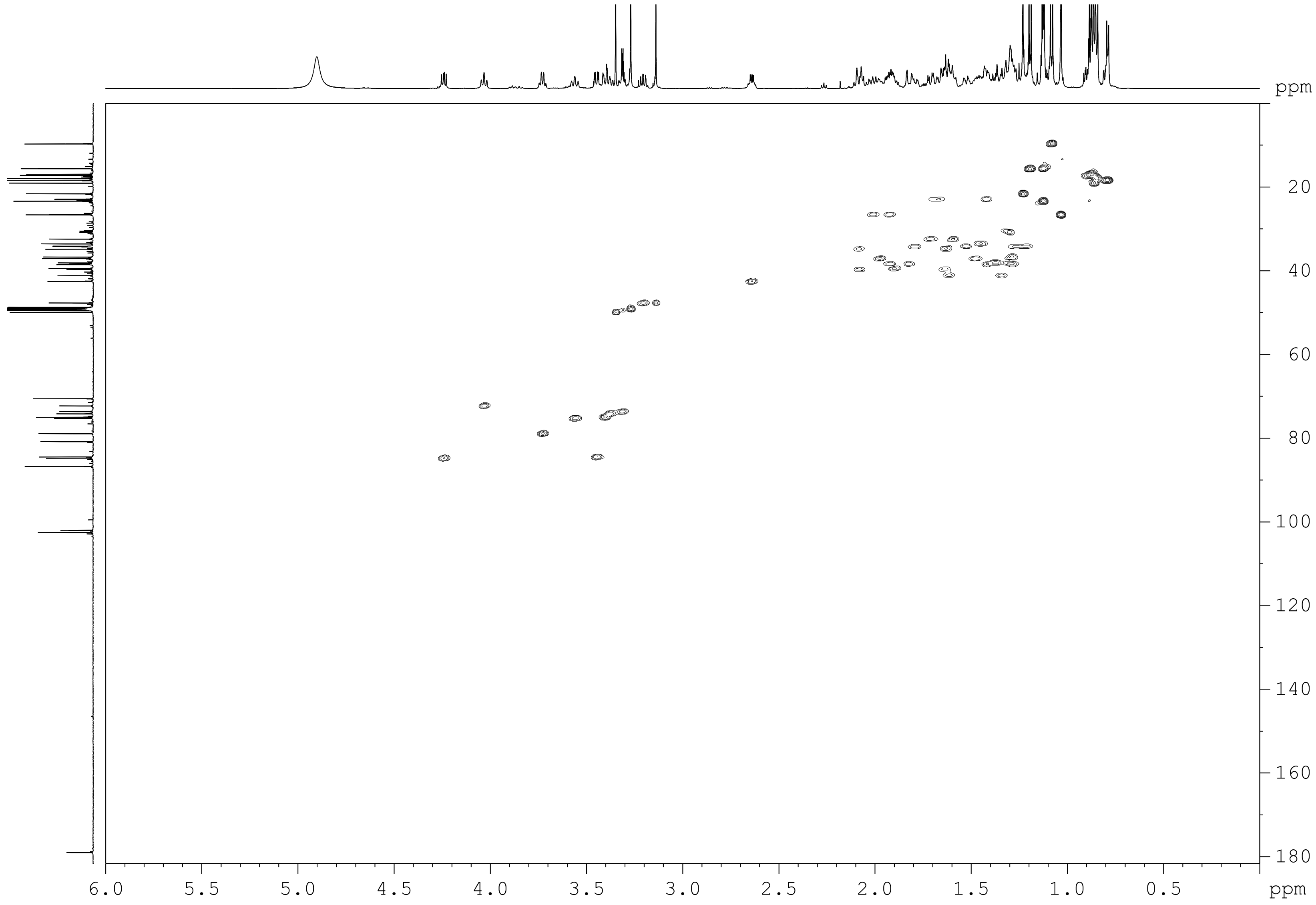

Figure S13. HSQC spectrum (^1H 600 MHz, ^{13}C 150 MHz, MeOD-d_4) of terrosamycin B (**2**).

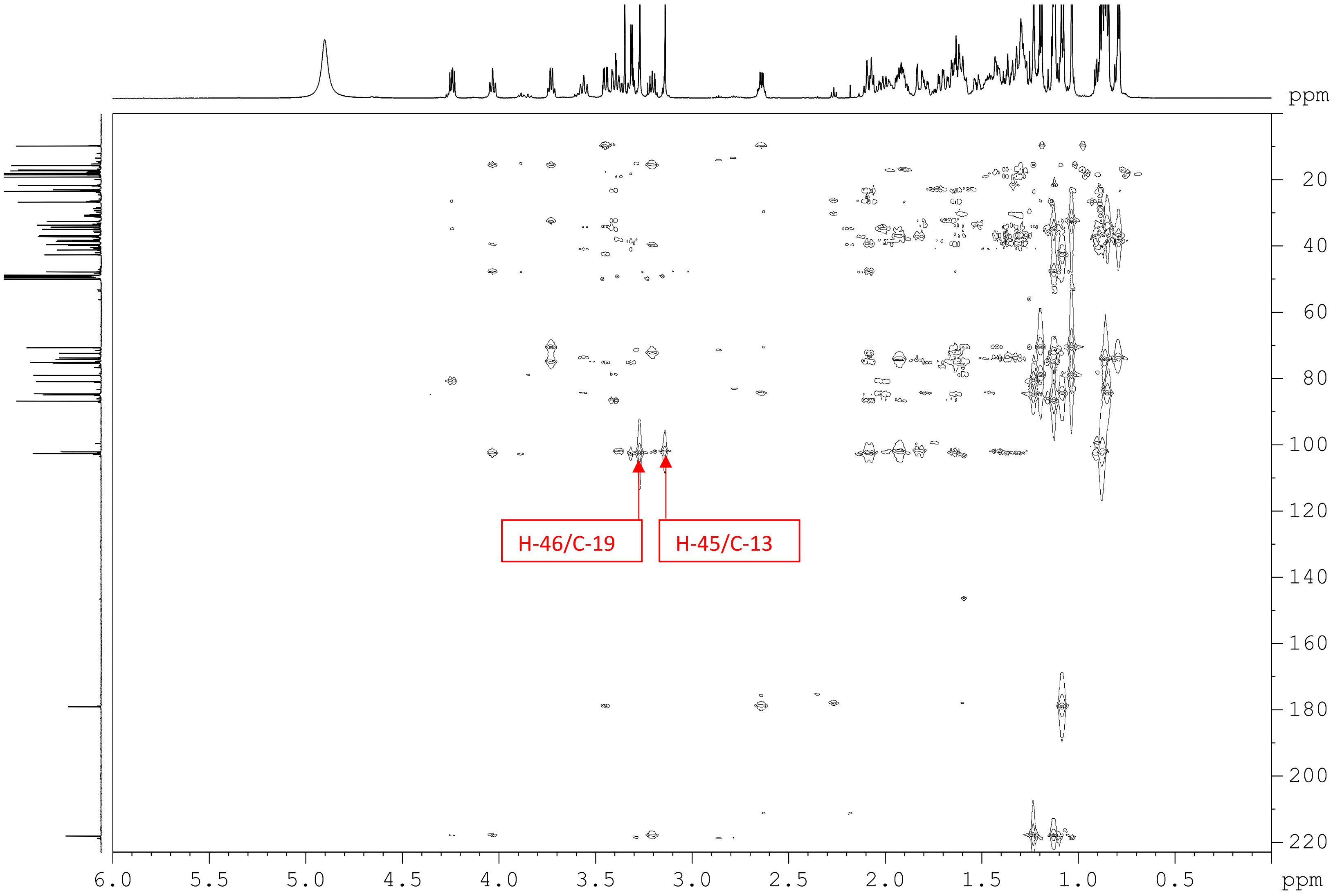

Figure S14. HMBC spectrum (^{1}H 600 MHz, ^{13}C 150 MHz, MeOD-d$_4$) of terrosamycin B (**2**).

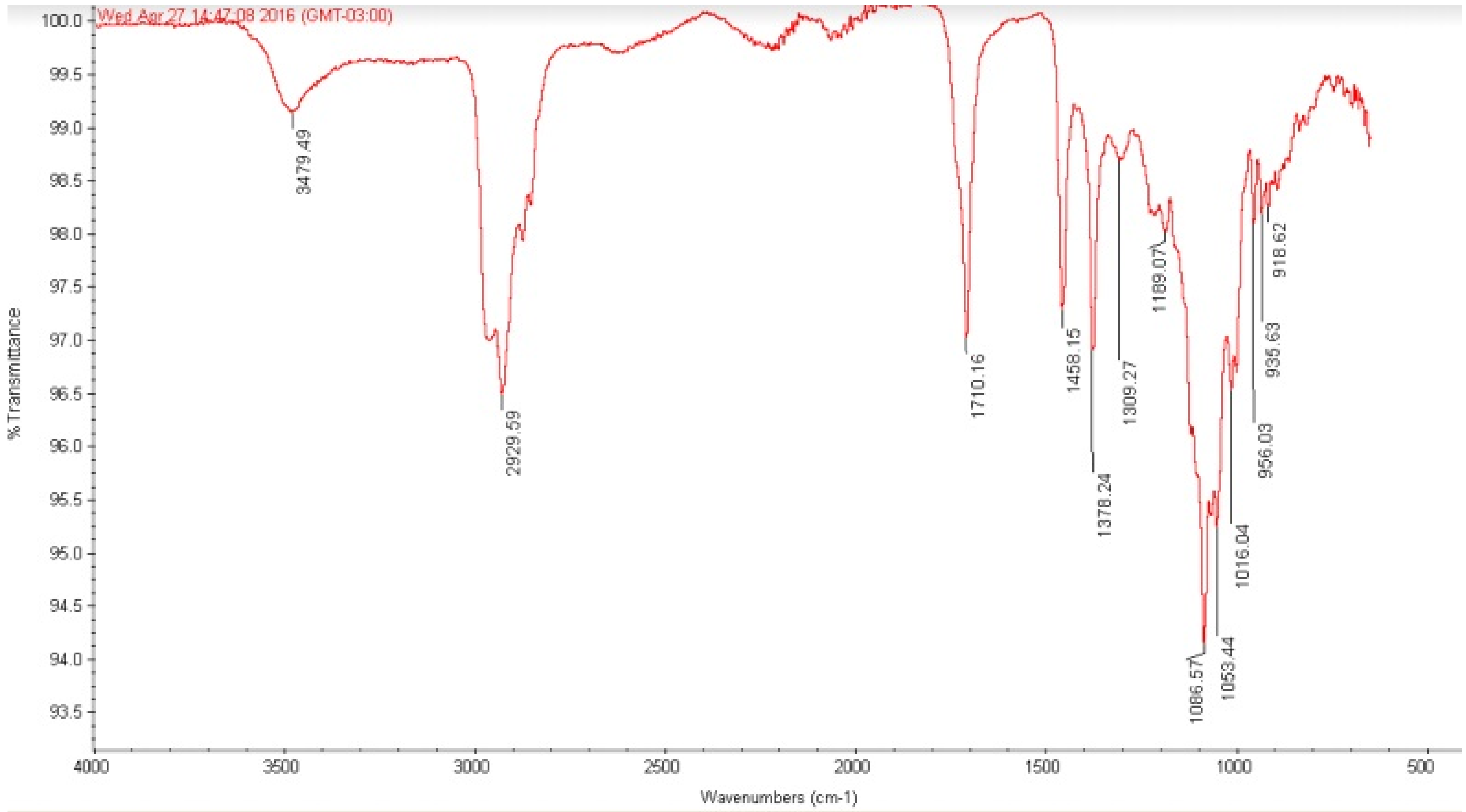

Figure S15. IR spectrum of terrosamycin B (**2**) (MeOH, film).

A

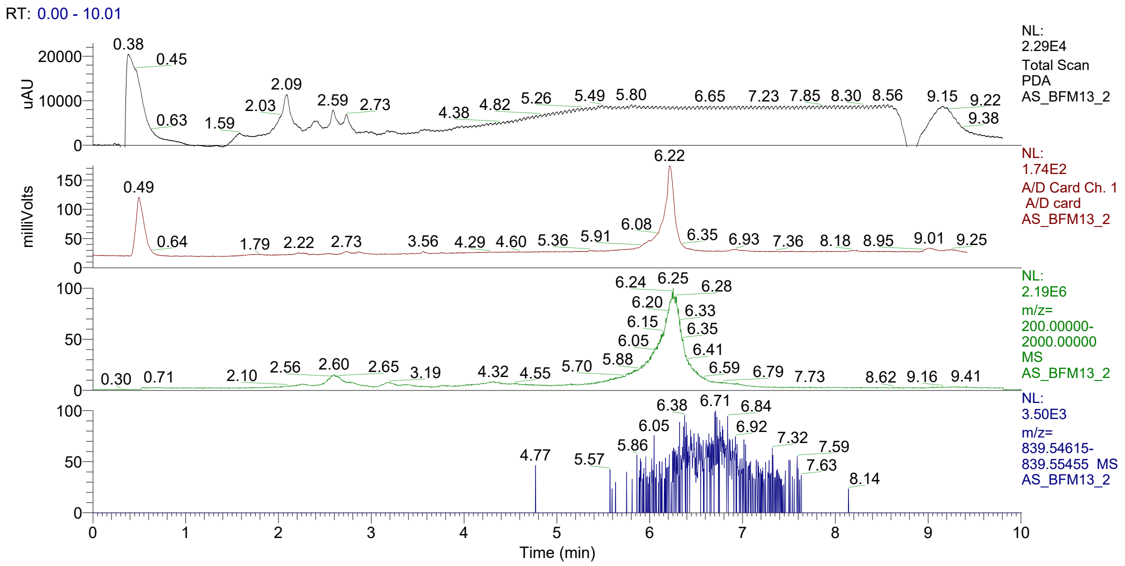

B

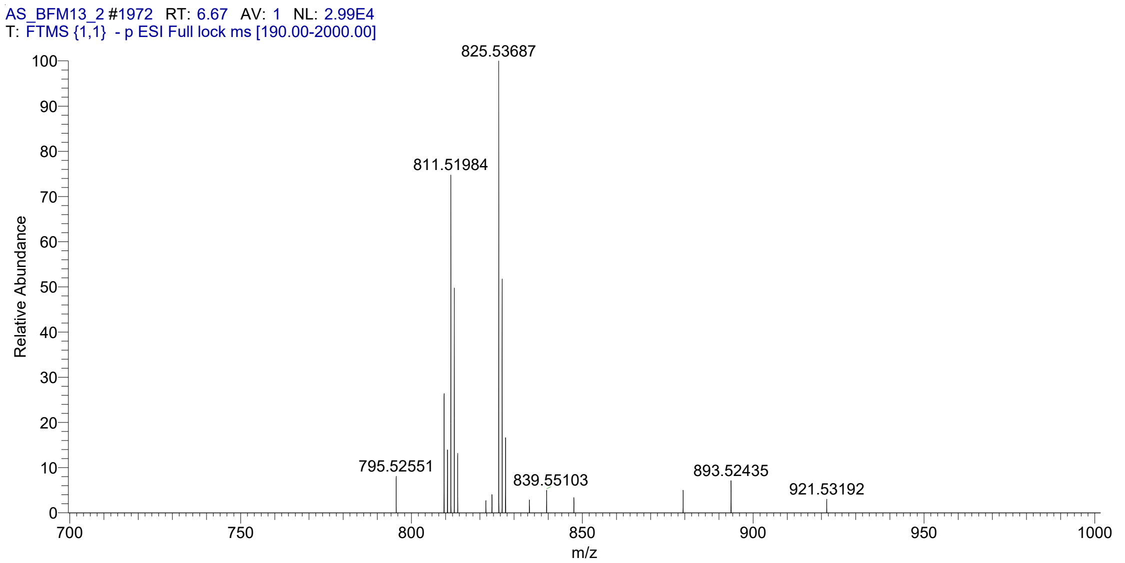

Figure S16. HPLC chromatograms of the crude acetonitrile extract of *Streptomyces* sp. RKND004 grown in BFM13. The top chromatogram was generated using a PDA detector, the second using an ELSD, and the third using –ESI-HRMS. The bottom trace is an extracted ion chromatogram monitoring *m/z* 839.5504 [M-H]$^-$, showing that terrosamycin B (**2**) is a true natural product and not only the result of methylation during sample preparation. **B**. –ESI-HRMS spectrum of the eluent at 6.7 min.

Table S2. Crystal data and structure refinement for terrosamycin A (**1**).

Identification code	RK160391	
Empirical formula	C45.50 H79 K O14	
Moiety formula	$C_{44}H_{75}KO_{13}$ • ½ Me_2CO • ½ H_2O	
Formula weight	889.19	
Temperature	173(1) K	
Wavelength	0.71073 Å	
Diffractometer used	Bruker AXS P4/SMART 1000	
Detector distance	5 cm	
Monochromator used	Graphite	
Crystal size	0.50 x 0.30 x 0.30 mm^3	
Colour and habit	Colourless, block	
Crystal system	Monoclinic	
Space group	P2(1)	
Unit cell dimensions	a = 12.004(3) Å	α = 90°
	b = 35.796(10) Å	β = 103.826(4)°
	c = 12.020(3) Å	γ = 90°
Volume	5015(2) $Å^3$	
Z	4	
Density (calculated)	1.178 Mg/m^3	
Absorption coefficient	0.166 mm^{-1}	
F(000)	1932	
Theta range for data collection	1.74 to 27.50°	
Completeness to theta = 25.00°	99.8 %	
Scan type	ω and ϕ	
Scan range	0.3°	

Exposure time	10s
Index ranges	$-15 \le h \le 14$, $-46 \le k \le 46$, $-15 \le l \le 13$
Standard reflections	50 frames at beginning and end of data collection
Crystal stability	no decay
Reflections collected	34091
Independent reflections	21746 [R(int) = 0.0760]
Solution	Direct methods
Hydrogen atoms	Mixed
Absorption correction	SADABS
Max. and min. transmission	0.9520 and 0.9217
Refinement method	Full-matrix least-squares on F^2
Data / restraints / parameters	21746 / 25 / 1158
Goodness-of-fit on F^2	1.019
Final R indices [I>2sigma(I)]	R1 = 0.0781, wR2 = 0.1906
R indices (all data)	R1 = 0.1075, wR2 = 0.2045
Largest/mean shift/esd	0.001/0.000
Absolute structure parameter	0.05(4)
Largest diff. peak and hole	0.938 and -0.343 e.Å^{-3}

$wR2 = (\sum[w(F_o^2-F_c^2)^2]/\sum[wF_o^4])^{1/2}$

$R1 = \sum||F_o| - |F_c||/\sum|F_o|$

Weight = $1 / [\sigma^2(F_o^2) + (0.0949 * P)^2]$

where $P = (\max(F_o^2, 0) + 2 * F_c^2)/3$

Table S3. Atomic coordinates ($x10^4$) and equivalent isotropic displacement parameters ($Å^2x10^3$) for terrosamycin A (**1**). U(eq) is defined as one third of the trace of the orthogonalized U^{ij} tensor.

	x	y	z	U(eq)
K(1)	7586(1)	7727(1)	8471(1)	36(1)
O(1)	9123(3)	7802(1)	10852(2)	35(1)
O(2)	8557(3)	8315(1)	9880(2)	44(1)
O(3)	11387(3)	8282(1)	11225(2)	36(1)
O(4)	11787(3)	7528(1)	9794(2)	35(1)
O(5)	9813(3)	7469(1)	9143(2)	36(1)
O(6)	9765(3)	6922(1)	11086(2)	32(1)
O(7)	9702(3)	7326(1)	12596(2)	36(1)
O(8)	7679(3)	7034(1)	9796(2)	40(1)
O(9)	5530(3)	7572(1)	9446(3)	53(1)
O(10)	5723(3)	6657(1)	8587(3)	52(1)
O(11)	5795(3)	7277(1)	7182(2)	45(1)
O(12)	7355(3)	7592(1)	5999(2)	42(1)
O(13)	7543(4)	8291(1)	6989(2)	51(1)
C(1)	9070(4)	8156(1)	10759(3)	33(1)
C(2)	9672(4)	8383(1)	11825(3)	38(1)
C(3)	10902(5)	8253(1)	12199(3)	41(1)
C(4)	11683(5)	8456(2)	13216(4)	55(2)
C(5)	12892(6)	8305(2)	13430(5)	67(2)
C(6)	13336(5)	8307(2)	12338(5)	61(2)
C(7)	12474(4)	8110(2)	11381(4)	44(1)
C(8)	12781(4)	8095(2)	10234(4)	48(1)
C(9)	11877(4)	7897(1)	9348(3)	36(1)

C(10)	12121(6)	7854(2)	8160(4)	55(1)
C(11)	11169(5)	7628(2)	7425(4)	57(2)
C(12)	10955(5)	7247(2)	7931(3)	48(1)
C(13)	10870(4)	7305(1)	9194(3)	33(1)
C(14)	11041(4)	6936(1)	9864(3)	32(1)
C(15)	10942(4)	6972(1)	11119(3)	32(1)
C(16)	11693(4)	6699(2)	11932(4)	44(1)
C(17)	11377(5)	6736(1)	13105(3)	45(1)
C(18)	10116(5)	6690(1)	13028(3)	41(1)
C(19)	9420(4)	6967(1)	12159(3)	35(1)
C(20)	8153(4)	6886(1)	11848(3)	40(1)
C(21)	7434(4)	7126(1)	10891(3)	35(1)
C(22)	6147(4)	7070(2)	10737(4)	44(1)
C(23)	5564(4)	7237(1)	9584(4)	40(1)
C(24)	5011(4)	6970(2)	8617(4)	45(1)
C(25)	4746(4)	7170(2)	7465(4)	46(1)
C(26)	4088(5)	6942(2)	6446(5)	66(2)
C(27)	4477(5)	7115(2)	5463(5)	69(2)
C(28)	5735(5)	7210(2)	5972(4)	48(1)
C(29)	6166(4)	7560(1)	5484(3)	41(1)
C(30)	5554(4)	7922(2)	5628(4)	50(1)
C(31)	6115(5)	8255(2)	5221(4)	54(1)
C(32)	7411(5)	8265(1)	5759(3)	45(1)
C(33)	7935(5)	7897(1)	5557(4)	44(1)
C(34)	8033(7)	7815(2)	4343(5)	69(2)
C(35)	7995(6)	8599(2)	5370(4)	58(2)
C(36)	6512(6)	6880(2)	5910(5)	63(2)

C(37)	3917(5)	6830(2)	8914(5)	57(1)
C(38)	5707(5)	7263(2)	11698(5)	64(2)
C(39)	9865(6)	6733(2)	14221(4)	56(2)
C(40)	12956(5)	6761(2)	12066(4)	62(2)
C(41)	9931(6)	7051(2)	7211(4)	61(2)
C(42)	12268(8)	8236(2)	7616(5)	85(2)
C(43)	11205(7)	8416(2)	14298(4)	80(2)
C(44)	9542(5)	8801(1)	11637(4)	47(1)
K(2)	1494(1)	5005(1)	598(1)	40(1)
O(101)	2901(3)	4399(1)	1518(3)	49(1)
O(102)	3856(3)	4911(1)	2142(2)	36(1)
O(103)	4299(3)	4409(1)	4377(2)	37(1)
O(104)	2872(3)	5146(1)	4862(2)	32(1)
O(105)	2123(3)	5198(1)	2893(2)	33(1)
O(106)	4107(3)	5786(1)	2816(2)	30(1)
O(107)	5594(3)	5378(1)	2701(3)	38(1)
O(108)	2827(3)	5704(1)	738(2)	42(1)
O(109)	2418(4)	5176(1)	-1443(3)	68(1)
O(110)	1715(4)	6108(1)	-1166(3)	50(1)
O(111)	231(3)	5502(1)	-1078(3)	53(1)
O(112)	-938(3)	5152(1)	440(3)	44(1)
O(113)	-35(3)	4446(1)	421(3)	49(1)
C(101)	3781(4)	4560(1)	2047(3)	35(1)
C(102)	4850(4)	4334(1)	2627(4)	38(1)
C(103)	5262(4)	4458(1)	3879(4)	41(1)
C(104)	6311(4)	4260(2)	4582(5)	52(1)
C(105)	6565(5)	4401(2)	5823(5)	70(2)

C(106)	5501(5)	4387(2)	6298(4)	58(2)
C(107)	4500(5)	4582(1)	5487(3)	44(1)
C(108)	3377(5)	4583(1)	5847(3)	41(1)
C(109)	2423(4)	4777(1)	4998(3)	36(1)
C(110)	1297(5)	4821(2)	5332(4)	49(1)
C(111)	498(4)	5046(2)	4386(4)	48(1)
C(112)	996(4)	5414(1)	4123(4)	42(1)
C(113)	2209(4)	5369(1)	3981(3)	31(1)
C(114)	2889(4)	5734(1)	4088(3)	31(1)
C(115)	4119(4)	5695(1)	4007(3)	31(1)
C(116)	4973(4)	5945(1)	4790(3)	37(1)
C(117)	6136(4)	5926(2)	4500(4)	44(1)
C(118)	6082(4)	6008(1)	3244(4)	42(1)
C(119)	5183(4)	5745(1)	2510(3)	33(1)
C(120)	4884(4)	5842(1)	1210(4)	42(1)
C(121)	3918(4)	5610(1)	509(3)	41(1)
C(122)	3785(5)	5662(2)	-793(3)	46(1)
C(123)	2599(5)	5514(2)	-1380(3)	49(1)
C(124)	1665(6)	5787(2)	-1898(3)	53(1)
C(125)	493(6)	5615(2)	-2164(4)	60(2)
C(126)	-502(7)	5869(2)	-2722(6)	87(2)
C(127)	-1505(7)	5697(2)	-2343(6)	89(2)
C(128)	-935(5)	5574(2)	-1097(4)	53(1)
C(129)	-1483(5)	5215(2)	-749(4)	53(1)
C(130)	-1389(6)	4870(2)	-1445(4)	59(2)
C(131)	-1819(5)	4529(2)	-949(5)	59(2)
C(132)	-1236(5)	4479(1)	346(4)	45(1)

C(133)	-1395(4)	4844(1)	969(4)	46(1)
C(134)	-2593(6)	4922(2)	1074(7)	78(2)
C(135)	-1689(5)	4138(2)	849(6)	63(2)
C(136)	-989(6)	5887(2)	-242(7)	77(2)
C(137)	1940(8)	5937(2)	-3007(4)	77(2)
C(138)	4715(6)	5447(2)	-1191(4)	70(2)
C(139)	7244(5)	5963(2)	2988(6)	61(2)
C(140)	5109(5)	5832(2)	6066(4)	54(1)
C(141)	201(5)	5610(2)	3094(5)	56(1)
C(142)	757(6)	4446(2)	5540(6)	71(2)
C(143)	7351(5)	4314(2)	4070(7)	81(2)
C(144)	4680(5)	3909(1)	2484(4)	53(1)
O(201)	8691(3)	8870(1)	8384(3)	45(1)
O(202)	6548(10)	8710(3)	2504(6)	126(3)
C(201)	6202(10)	8755(3)	1511(7)	70(3)
C(202)	6056(11)	8442(3)	690(9)	78(3)
C(203)	6030(20)	9134(4)	998(19)	138(8)
O(302)	4550(20)	8989(7)	542(15)	126(3)
C(301)	5550(20)	8969(7)	830(20)	70(3)
C(302)	6040(30)	8593(6)	1120(30)	78(3)
C(303)	6350(50)	9288(8)	1000(60)	138(8)

Table S4. Bond lengths [Å] and angles [°] for terrosamycin A (**1**).

K(1)-O(13)	2.683(3)
K(1)-O(5)	2.761(3)
K(1)-O(2)	2.778(3)
K(1)-O(11)	2.833(3)
K(1)-O(8)	2.935(3)
K(1)-O(12)	2.957(3)
K(1)-O(1)	3.024(3)
K(1)-O(9)	3.025(4)
K(1)-H(5)	2.26(13)
K(1)-H(13)	2.95(13)
O(1)-C(1)	1.274(5)
O(2)-C(1)	1.229(5)
O(3)-C(7)	1.415(6)
O(3)-C(3)	1.431(5)
O(4)-C(13)	1.411(5)
O(4)-C(9)	1.439(5)
O(5)-C(13)	1.387(5)
O(5)-H(5)	0.81(6)
O(6)-C(15)	1.415(5)
O(6)-C(19)	1.455(4)
O(7)-C(19)	1.399(5)
O(7)-H(7)	0.8400
O(8)-C(21)	1.453(5)
O(8)-H(8)	0.81(6)
O(9)-C(23)	1.211(6)

O(10)-C(24)	1.415(6)
O(10)-H(10)	0.8400
O(11)-C(25)	1.433(6)
O(11)-C(28)	1.460(5)
O(12)-C(29)	1.418(6)
O(12)-C(33)	1.460(6)
O(13)-C(32)	1.452(5)
O(13)-H(13)	0.81(6)
C(1)-C(2)	1.543(6)
C(2)-C(3)	1.511(7)
C(2)-C(44)	1.516(7)
C(2)-H(2)	1.0000
C(3)-C(4)	1.534(6)
C(3)-H(3)	1.0000
C(4)-C(5)	1.513(9)
C(4)-C(43)	1.548(8)
C(4)-H(4)	1.0000
C(5)-C(6)	1.530(9)
C(5)-H(5A)	0.9900
C(5)-H(5B)	0.9900
C(6)-C(7)	1.524(7)
C(6)-H(6A)	0.9900
C(6)-H(6B)	0.9900
C(7)-C(8)	1.511(7)
C(7)-H(7A)	1.0000
C(8)-C(9)	1.503(7)
C(8)-H(8A)	0.9900

C(8)-H(8B)	0.9900
C(9)-C(10)	1.533(6)
C(9)-H(9)	1.0000
C(10)-C(11)	1.501(9)
C(10)-C(42)	1.547(8)
C(10)-H(10A)	1.0000
C(11)-C(12)	1.541(8)
C(11)-H(11A)	0.9900
C(11)-H(11B)	0.9900
C(12)-C(41)	1.499(8)
C(12)-C(13)	1.559(5)
C(12)-H(12)	1.0000
C(13)-C(14)	1.535(6)
C(14)-C(15)	1.547(5)
C(14)-H(14A)	0.9900
C(14)-H(14B)	0.9900
C(15)-C(16)	1.515(6)
C(15)-H(15)	1.0000
C(16)-C(40)	1.502(8)
C(16)-C(17)	1.550(6)
C(16)-H(16)	1.0000
C(17)-C(18)	1.503(7)
C(17)-H(17A)	0.9900
C(17)-H(17B)	0.9900
C(18)-C(19)	1.535(6)
C(18)-C(39)	1.542(5)
C(18)-H(18)	1.0000

C(19)-C(20)	1.506(7)
C(20)-C(21)	1.525(7)
C(20)-H(20A)	0.9900
C(20)-H(20B)	0.9900
C(21)-C(22)	1.524(7)
C(21)-H(21)	1.0000
C(22)-C(23)	1.519(7)
C(22)-C(38)	1.544(7)
C(22)-H(22)	1.0000
C(23)-C(24)	1.530(7)
C(24)-C(25)	1.523(7)
C(24)-C(37)	1.526(7)
C(25)-C(26)	1.526(7)
C(25)-H(25)	1.0000
C(26)-C(27)	1.503(9)
C(26)-H(26A)	0.9900
C(26)-H(26B)	0.9900
C(27)-C(28)	1.526(8)
C(27)-H(27A)	0.9900
C(27)-H(27B)	0.9900
C(28)-C(36)	1.515(8)
C(28)-C(29)	1.528(7)
C(29)-C(30)	1.519(8)
C(29)-H(29)	1.0000
C(30)-C(31)	1.507(8)
C(30)-H(30A)	0.9900
C(30)-H(30B)	0.9900

C(31)-C(32)	1.536(8)
C(31)-H(31A)	0.9900
C(31)-H(31B)	0.9900
C(32)-C(33)	1.504(7)
C(32)-C(35)	1.516(8)
C(33)-C(34)	1.521(7)
C(33)-H(33)	1.0000
C(34)-H(34A)	0.9800
C(34)-H(34B)	0.9800
C(34)-H(34C)	0.9800
C(35)-H(35A)	0.9800
C(35)-H(35B)	0.9800
C(35)-H(35C)	0.9800
C(36)-H(36A)	0.9800
C(36)-H(36B)	0.9800
C(36)-H(36C)	0.9800
C(37)-H(37A)	0.9800
C(37)-H(37B)	0.9800
C(37)-H(37C)	0.9800
C(38)-H(38A)	0.9800
C(38)-H(38B)	0.9800
C(38)-H(38C)	0.9800
C(39)-H(39A)	0.9800
C(39)-H(39B)	0.9800
C(39)-H(39C)	0.9800
C(40)-H(40A)	0.9800
C(40)-H(40B)	0.9800

C(40)-H(40C)	0.9800
C(41)-H(41A)	0.9800
C(41)-H(41B)	0.9800
C(41)-H(41C)	0.9800
C(42)-H(42A)	0.9800
C(42)-H(42B)	0.9800
C(42)-H(42C)	0.9800
C(43)-H(43A)	0.9800
C(43)-H(43B)	0.9800
C(43)-H(43C)	0.9800
C(44)-H(44A)	0.9800
C(44)-H(44B)	0.9800
C(44)-H(44C)	0.9800
K(2)-O(113)	2.689(4)
K(2)-O(105)	2.768(3)
K(2)-O(101)	2.810(4)
K(2)-O(111)	2.838(3)
K(2)-O(112)	2.928(4)
K(2)-O(108)	2.953(4)
K(2)-O(109)	2.987(4)
K(2)-O(102)	3.013(3)
K(2)-H(105)	2.80(13)
K(2)-H(108)	2.31(11)
K(2)-H(113)	3.00(13)
O(101)-C(101)	1.238(6)
O(102)-C(101)	1.262(5)
O(103)-C(103)	1.435(6)

O(103)-C(107)	1.439(5)
O(104)-C(113)	1.411(5)
O(104)-C(109)	1.450(5)
O(105)-C(113)	1.426(4)
O(105)-H(105)	0.81(6)
O(106)-C(119)	1.432(5)
O(106)-C(115)	1.464(4)
O(107)-C(119)	1.403(5)
O(107)-H(107)	0.8400
O(108)-C(121)	1.441(6)
O(108)-H(108)	0.81(6)
O(109)-C(123)	1.231(6)
O(110)-C(124)	1.440(6)
O(110)-H(110)	0.8400
O(111)-C(128)	1.419(7)
O(111)-C(125)	1.471(6)
O(112)-C(129)	1.441(6)
O(112)-C(133)	1.446(6)
O(113)-C(132)	1.428(7)
O(113)-H(113)	0.81(6)
C(101)-C(102)	1.535(7)
C(102)-C(103)	1.532(6)
C(102)-C(144)	1.539(7)
C(102)-H(102)	1.0000
C(103)-C(104)	1.515(7)
C(103)-H(103)	1.0000
C(104)-C(143)	1.530(9)

C(104)-C(105)	1.536(8)
C(104)-H(104)	1.0000
C(105)-C(106)	1.519(10)
C(105)-H(10B)	0.9900
C(105)-H(10C)	0.9900
C(106)-C(107)	1.525(7)
C(106)-H(10D)	0.9900
C(106)-H(10E)	0.9900
C(107)-C(108)	1.510(7)
C(107)-H(10F)	1.0000
C(108)-C(109)	1.509(7)
C(108)-H(10G)	0.9900
C(108)-H(10H)	0.9900
C(109)-C(110)	1.508(7)
C(109)-H(109)	1.0000
C(110)-C(111)	1.529(8)
C(110)-C(142)	1.539(8)
C(110)-H(11C)	1.0000
C(111)-C(112)	1.513(7)
C(111)-H(11D)	0.9900
C(111)-H(11E)	0.9900
C(112)-C(113)	1.516(6)
C(112)-C(141)	1.538(8)
C(112)-H(112)	1.0000
C(113)-C(114)	1.527(6)
C(114)-C(115)	1.509(6)
C(114)-H(11F)	0.9900

C(114)-H(11G)	0.9900
C(115)-C(116)	1.508(6)
C(115)-H(115)	1.0000
C(116)-C(117)	1.520(7)
C(116)-C(140)	1.556(6)
C(116)-H(116)	1.0000
C(117)-C(118)	1.524(7)
C(117)-H(11H)	0.9900
C(117)-H(11I)	0.9900
C(118)-C(139)	1.506(7)
C(118)-C(119)	1.541(6)
C(118)-H(118)	1.0000
C(119)-C(120)	1.556(6)
C(120)-C(121)	1.510(7)
C(120)-H(12A)	0.9900
C(120)-H(12B)	0.9900
C(121)-C(122)	1.545(5)
C(121)-H(121)	1.0000
C(122)-C(123)	1.524(8)
C(122)-C(138)	1.526(8)
C(122)-H(122)	1.0000
C(123)-C(124)	1.505(8)
C(124)-C(125)	1.499(9)
C(124)-C(137)	1.545(6)
C(125)-C(126)	1.526(10)
C(125)-H(125)	1.0000
C(126)-C(127)	1.517(11)

C(126)-H(12C)	0.9900
C(126)-H(12D)	0.9900
C(127)-C(128)	1.553(8)
C(127)-H(12E)	0.9900
C(127)-H(12F)	0.9900
C(128)-C(136)	1.532(9)
C(128)-C(129)	1.546(8)
C(129)-C(130)	1.513(8)
C(129)-H(129)	1.0000
C(130)-C(131)	1.501(9)
C(130)-H(13A)	0.9900
C(130)-H(13B)	0.9900
C(131)-C(132)	1.558(7)
C(131)-H(13C)	0.9900
C(131)-H(13D)	0.9900
C(132)-C(135)	1.520(8)
C(132)-C(133)	1.538(7)
C(133)-C(134)	1.500(8)
C(133)-H(133)	1.0000
C(134)-H(13E)	0.9800
C(134)-H(13F)	0.9800
C(134)-H(13G)	0.9800
C(135)-H(13H)	0.9800
C(135)-H(13I)	0.9800
C(135)-H(13J)	0.9800
C(136)-H(13K)	0.9800
C(136)-H(13L)	0.9800

C(136)-H(13M)	0.9800
C(137)-H(13N)	0.9800
C(137)-H(13O)	0.9800
C(137)-H(13P)	0.9800
C(138)-H(13Q)	0.9800
C(138)-H(13R)	0.9800
C(138)-H(13S)	0.9800
C(139)-H(13T)	0.9800
C(139)-H(13U)	0.9800
C(139)-H(13V)	0.9800
C(140)-H(14C)	0.9800
C(140)-H(14D)	0.9800
C(140)-H(14E)	0.9800
C(141)-H(14F)	0.9800
C(141)-H(14G)	0.9800
C(141)-H(14H)	0.9800
C(142)-H(14I)	0.9800
C(142)-H(14J)	0.9800
C(142)-H(14K)	0.9800
C(143)-H(14L)	0.9800
C(143)-H(14M)	0.9800
C(143)-H(14N)	0.9800
C(144)-H(14O)	0.9800
C(144)-H(14P)	0.9800
C(144)-H(14Q)	0.9800
O(201)-H(201)	0.90(9)
O(201)-H(202)	0.90(9)

O(202)-C(201)	1.177(10)
C(201)-C(202)	1.476(10)
C(201)-C(203)	1.485(11)
C(202)-H(20C)	0.9800
C(202)-H(20D)	0.9800
C(202)-H(20E)	0.9800
C(203)-H(20F)	0.9800
C(203)-H(20G)	0.9800
C(203)-H(20H)	0.9800
O(302)-C(301)	1.173(14)
C(301)-C(302)	1.475(13)
C(301)-C(303)	1.475(13)
C(302)-H(30C)	0.9800
C(302)-H(30D)	0.9800
C(302)-H(30E)	0.9800
C(303)-H(30F)	0.9800
C(303)-H(30G)	0.9800
C(303)-H(30H)	0.9800

O(13)-K(1)-O(5)	108.01(12)
O(13)-K(1)-O(2)	76.92(10)
O(5)-K(1)-O(2)	80.75(10)
O(13)-K(1)-O(11)	99.96(11)
O(5)-K(1)-O(11)	121.65(11)
O(2)-K(1)-O(11)	156.58(11)
O(13)-K(1)-O(8)	170.94(10)
O(5)-K(1)-O(8)	69.48(9)

O(2)-K(1)-O(8)	110.70(9)
O(11)-K(1)-O(8)	75.04(10)
O(13)-K(1)-O(12)	58.22(10)
O(5)-K(1)-O(12)	95.21(9)
O(2)-K(1)-O(12)	131.40(10)
O(11)-K(1)-O(12)	58.73(9)
O(8)-K(1)-O(12)	112.92(9)
O(13)-K(1)-O(1)	118.20(10)
O(5)-K(1)-O(1)	54.78(8)
O(2)-K(1)-O(1)	44.26(8)
O(11)-K(1)-O(1)	141.22(9)
O(8)-K(1)-O(1)	68.00(8)
O(12)-K(1)-O(1)	148.74(9)
O(13)-K(1)-O(9)	120.47(12)
O(5)-K(1)-O(9)	130.38(10)
O(2)-K(1)-O(9)	99.50(10)
O(11)-K(1)-O(9)	61.65(10)
O(8)-K(1)-O(9)	64.16(10)
O(12)-K(1)-O(9)	118.04(10)
O(1)-K(1)-O(9)	91.11(9)
O(13)-K(1)-H(5)	93(2)
O(5)-K(1)-H(5)	15(2)
O(2)-K(1)-H(5)	77(4)
O(11)-K(1)-H(5)	126(4)
O(8)-K(1)-H(5)	84(2)
O(12)-K(1)-H(5)	87(3)
O(1)-K(1)-H(5)	62(3)

O(9)-K(1)-H(5)	145(2)
O(13)-K(1)-H(13)	15.6(16)
O(5)-K(1)-H(13)	106(3)
O(2)-K(1)-H(13)	61.5(17)
O(11)-K(1)-H(13)	113(2)
O(8)-K(1)-H(13)	172(2)
O(12)-K(1)-H(13)	73.7(17)
O(1)-K(1)-H(13)	104(2)
O(9)-K(1)-H(13)	118(3)
H(5)-K(1)-H(13)	92(4)
C(1)-O(1)-K(1)	89.9(2)
C(1)-O(2)-K(1)	102.9(3)
C(7)-O(3)-C(3)	113.7(3)
C(13)-O(4)-C(9)	116.6(3)
C(13)-O(5)-K(1)	165.2(2)
C(13)-O(5)-H(5)	128(10)
K(1)-O(5)-H(5)	45(10)
C(15)-O(6)-C(19)	117.0(3)
C(19)-O(7)-H(7)	109.5
C(21)-O(8)-K(1)	108.2(3)
C(21)-O(8)-H(8)	93(10)
K(1)-O(8)-H(8)	99(10)
C(23)-O(9)-K(1)	103.3(3)
C(24)-O(10)-H(10)	109.5
C(25)-O(11)-C(28)	110.8(3)
C(25)-O(11)-K(1)	128.2(3)
C(28)-O(11)-K(1)	119.3(3)

C(29)-O(12)-C(33)	114.8(4)
C(29)-O(12)-K(1)	107.2(2)
C(33)-O(12)-K(1)	107.8(2)
C(32)-O(13)-K(1)	127.5(3)
C(32)-O(13)-H(13)	131(10)
K(1)-O(13)-H(13)	101(10)
O(2)-C(1)-O(1)	122.8(4)
O(2)-C(1)-C(2)	120.6(4)
O(1)-C(1)-C(2)	116.6(4)
C(3)-C(2)-C(44)	114.0(4)
C(3)-C(2)-C(1)	108.4(4)
C(44)-C(2)-C(1)	112.6(4)
C(3)-C(2)-H(2)	107.2
C(44)-C(2)-H(2)	107.2
C(1)-C(2)-H(2)	107.2
O(3)-C(3)-C(2)	107.1(3)
O(3)-C(3)-C(4)	109.1(4)
C(2)-C(3)-C(4)	117.3(4)
O(3)-C(3)-H(3)	107.7
C(2)-C(3)-H(3)	107.7
C(4)-C(3)-H(3)	107.7
C(5)-C(4)-C(3)	110.2(5)
C(5)-C(4)-C(43)	111.0(5)
C(3)-C(4)-C(43)	110.3(5)
C(5)-C(4)-H(4)	108.4
C(3)-C(4)-H(4)	108.4
C(43)-C(4)-H(4)	108.4

C(4)-C(5)-C(6)	111.7(4)
C(4)-C(5)-H(5A)	109.3
C(6)-C(5)-H(5A)	109.3
C(4)-C(5)-H(5B)	109.3
C(6)-C(5)-H(5B)	109.3
H(5A)-C(5)-H(5B)	107.9
C(7)-C(6)-C(5)	109.4(5)
C(7)-C(6)-H(6A)	109.8
C(5)-C(6)-H(6A)	109.8
C(7)-C(6)-H(6B)	109.8
C(5)-C(6)-H(6B)	109.8
H(6A)-C(6)-H(6B)	108.2
O(3)-C(7)-C(8)	108.2(4)
O(3)-C(7)-C(6)	109.7(4)
C(8)-C(7)-C(6)	115.8(5)
O(3)-C(7)-H(7A)	107.6
C(8)-C(7)-H(7A)	107.6
C(6)-C(7)-H(7A)	107.6
C(9)-C(8)-C(7)	111.7(4)
C(9)-C(8)-H(8A)	109.3
C(7)-C(8)-H(8A)	109.3
C(9)-C(8)-H(8B)	109.3
C(7)-C(8)-H(8B)	109.3
H(8A)-C(8)-H(8B)	107.9
O(4)-C(9)-C(8)	105.9(3)
O(4)-C(9)-C(10)	107.6(4)
C(8)-C(9)-C(10)	116.1(4)

O(4)-C(9)-H(9)	109.0
C(8)-C(9)-H(9)	109.0
C(10)-C(9)-H(9)	109.0
C(11)-C(10)-C(9)	108.3(4)
C(11)-C(10)-C(42)	112.0(5)
C(9)-C(10)-C(42)	111.8(5)
C(11)-C(10)-H(10A)	108.2
C(9)-C(10)-H(10A)	108.2
C(42)-C(10)-H(10A)	108.2
C(10)-C(11)-C(12)	114.8(4)
C(10)-C(11)-H(11A)	108.6
C(12)-C(11)-H(11A)	108.6
C(10)-C(11)-H(11B)	108.6
C(12)-C(11)-H(11B)	108.6
H(11A)-C(11)-H(11B)	107.6
C(41)-C(12)-C(11)	112.2(4)
C(41)-C(12)-C(13)	112.9(4)
C(11)-C(12)-C(13)	108.8(4)
C(41)-C(12)-H(12)	107.5
C(11)-C(12)-H(12)	107.5
C(13)-C(12)-H(12)	107.5
O(5)-C(13)-O(4)	112.1(4)
O(5)-C(13)-C(14)	113.2(3)
O(4)-C(13)-C(14)	103.2(3)
O(5)-C(13)-C(12)	106.6(3)
O(4)-C(13)-C(12)	110.4(4)
C(14)-C(13)-C(12)	111.5(4)

C(13)-C(14)-C(15)	114.1(3)
C(13)-C(14)-H(14A)	108.7
C(15)-C(14)-H(14A)	108.7
C(13)-C(14)-H(14B)	108.7
C(15)-C(14)-H(14B)	108.7
H(14A)-C(14)-H(14B)	107.6
O(6)-C(15)-C(16)	111.9(3)
O(6)-C(15)-C(14)	105.7(3)
C(16)-C(15)-C(14)	113.8(4)
O(6)-C(15)-H(15)	108.4
C(16)-C(15)-H(15)	108.4
C(14)-C(15)-H(15)	108.4
C(40)-C(16)-C(15)	113.9(4)
C(40)-C(16)-C(17)	110.5(4)
C(15)-C(16)-C(17)	107.1(4)
C(40)-C(16)-H(16)	108.4
C(15)-C(16)-H(16)	108.4
C(17)-C(16)-H(16)	108.4
C(18)-C(17)-C(16)	113.4(4)
C(18)-C(17)-H(17A)	108.9
C(16)-C(17)-H(17A)	108.9
C(18)-C(17)-H(17B)	108.9
C(16)-C(17)-H(17B)	108.9
H(17A)-C(17)-H(17B)	107.7
C(17)-C(18)-C(19)	110.3(4)
C(17)-C(18)-C(39)	110.5(4)
C(19)-C(18)-C(39)	111.9(4)

C(17)-C(18)-H(18)	108.0
C(19)-C(18)-H(18)	108.0
C(39)-C(18)-H(18)	108.0
O(7)-C(19)-O(6)	109.9(3)
O(7)-C(19)-C(20)	114.2(4)
O(6)-C(19)-C(20)	104.1(3)
O(7)-C(19)-C(18)	107.1(3)
O(6)-C(19)-C(18)	107.9(3)
C(20)-C(19)-C(18)	113.4(4)
C(19)-C(20)-C(21)	115.8(4)
C(19)-C(20)-H(20A)	108.3
C(21)-C(20)-H(20A)	108.3
C(19)-C(20)-H(20B)	108.3
C(21)-C(20)-H(20B)	108.3
H(20A)-C(20)-H(20B)	107.4
O(8)-C(21)-C(22)	106.2(3)
O(8)-C(21)-C(20)	110.9(4)
C(22)-C(21)-C(20)	113.1(4)
O(8)-C(21)-H(21)	108.8
C(22)-C(21)-H(21)	108.8
C(20)-C(21)-H(21)	108.8
C(23)-C(22)-C(21)	107.0(3)
C(23)-C(22)-C(38)	109.9(5)
C(21)-C(22)-C(38)	111.6(4)
C(23)-C(22)-H(22)	109.4
C(21)-C(22)-H(22)	109.4
C(38)-C(22)-H(22)	109.4

O(9)-C(23)-C(22)	120.6(4)
O(9)-C(23)-C(24)	121.5(4)
C(22)-C(23)-C(24)	117.9(4)
O(10)-C(24)-C(25)	110.2(4)
O(10)-C(24)-C(37)	107.7(4)
C(25)-C(24)-C(37)	111.3(4)
O(10)-C(24)-C(23)	111.1(4)
C(25)-C(24)-C(23)	110.8(4)
C(37)-C(24)-C(23)	105.6(4)
O(11)-C(25)-C(24)	109.7(4)
O(11)-C(25)-C(26)	105.6(4)
C(24)-C(25)-C(26)	115.7(5)
O(11)-C(25)-H(25)	108.5
C(24)-C(25)-H(25)	108.5
C(26)-C(25)-H(25)	108.5
C(27)-C(26)-C(25)	102.2(5)
C(27)-C(26)-H(26A)	111.3
C(25)-C(26)-H(26A)	111.3
C(27)-C(26)-H(26B)	111.3
C(25)-C(26)-H(26B)	111.3
H(26A)-C(26)-H(26B)	109.2
C(26)-C(27)-C(28)	103.9(4)
C(26)-C(27)-H(27A)	111.0
C(28)-C(27)-H(27A)	111.0
C(26)-C(27)-H(27B)	111.0
C(28)-C(27)-H(27B)	111.0
H(27A)-C(27)-H(27B)	109.0

O(11)-C(28)-C(36)	107.1(4)
O(11)-C(28)-C(27)	104.1(4)
C(36)-C(28)-C(27)	111.6(5)
O(11)-C(28)-C(29)	107.8(4)
C(36)-C(28)-C(29)	110.6(4)
C(27)-C(28)-C(29)	115.0(5)
O(12)-C(29)-C(30)	110.2(4)
O(12)-C(29)-C(28)	107.2(4)
C(30)-C(29)-C(28)	116.1(4)
O(12)-C(29)-H(29)	107.7
C(30)-C(29)-H(29)	107.7
C(28)-C(29)-H(29)	107.7
C(31)-C(30)-C(29)	111.8(4)
C(31)-C(30)-H(30A)	109.3
C(29)-C(30)-H(30A)	109.3
C(31)-C(30)-H(30B)	109.3
C(29)-C(30)-H(30B)	109.3
H(30A)-C(30)-H(30B)	107.9
C(30)-C(31)-C(32)	111.6(4)
C(30)-C(31)-H(31A)	109.3
C(32)-C(31)-H(31A)	109.3
C(30)-C(31)-H(31B)	109.3
C(32)-C(31)-H(31B)	109.3
H(31A)-C(31)-H(31B)	108.0
O(13)-C(32)-C(33)	105.8(4)
O(13)-C(32)-C(35)	108.5(4)
C(33)-C(32)-C(35)	113.6(5)

O(13)-C(32)-C(31)	106.5(4)
C(33)-C(32)-C(31)	109.4(4)
C(35)-C(32)-C(31)	112.6(4)
O(12)-C(33)-C(32)	109.9(4)
O(12)-C(33)-C(34)	111.0(4)
C(32)-C(33)-C(34)	117.0(4)
O(12)-C(33)-H(33)	106.0
C(32)-C(33)-H(33)	106.0
C(34)-C(33)-H(33)	106.0
C(33)-C(34)-H(34A)	109.5
C(33)-C(34)-H(34B)	109.5
H(34A)-C(34)-H(34B)	109.5
C(33)-C(34)-H(34C)	109.5
H(34A)-C(34)-H(34C)	109.5
H(34B)-C(34)-H(34C)	109.5
C(32)-C(35)-H(35A)	109.5
C(32)-C(35)-H(35B)	109.5
H(35A)-C(35)-H(35B)	109.5
C(32)-C(35)-H(35C)	109.5
H(35A)-C(35)-H(35C)	109.5
H(35B)-C(35)-H(35C)	109.5
C(28)-C(36)-H(36A)	109.5
C(28)-C(36)-H(36B)	109.5
H(36A)-C(36)-H(36B)	109.5
C(28)-C(36)-H(36C)	109.5
H(36A)-C(36)-H(36C)	109.5
H(36B)-C(36)-H(36C)	109.5

C(24)-C(37)-H(37A)	109.5
C(24)-C(37)-H(37B)	109.5
H(37A)-C(37)-H(37B)	109.5
C(24)-C(37)-H(37C)	109.5
H(37A)-C(37)-H(37C)	109.5
H(37B)-C(37)-H(37C)	109.5
C(22)-C(38)-H(38A)	109.5
C(22)-C(38)-H(38B)	109.5
H(38A)-C(38)-H(38B)	109.5
C(22)-C(38)-H(38C)	109.5
H(38A)-C(38)-H(38C)	109.5
H(38B)-C(38)-H(38C)	109.5
C(18)-C(39)-H(39A)	109.5
C(18)-C(39)-H(39B)	109.5
H(39A)-C(39)-H(39B)	109.5
C(18)-C(39)-H(39C)	109.5
H(39A)-C(39)-H(39C)	109.5
H(39B)-C(39)-H(39C)	109.5
C(16)-C(40)-H(40A)	109.5
C(16)-C(40)-H(40B)	109.5
H(40A)-C(40)-H(40B)	109.5
C(16)-C(40)-H(40C)	109.5
H(40A)-C(40)-H(40C)	109.5
H(40B)-C(40)-H(40C)	109.5
C(12)-C(41)-H(41A)	109.5
C(12)-C(41)-H(41B)	109.5
H(41A)-C(41)-H(41B)	109.5

C(12)-C(41)-H(41C)	109.5
H(41A)-C(41)-H(41C)	109.5
H(41B)-C(41)-H(41C)	109.5
C(10)-C(42)-H(42A)	109.5
C(10)-C(42)-H(42B)	109.5
H(42A)-C(42)-H(42B)	109.5
C(10)-C(42)-H(42C)	109.5
H(42A)-C(42)-H(42C)	109.5
H(42B)-C(42)-H(42C)	109.5
C(4)-C(43)-H(43A)	109.5
C(4)-C(43)-H(43B)	109.5
H(43A)-C(43)-H(43B)	109.5
C(4)-C(43)-H(43C)	109.5
H(43A)-C(43)-H(43C)	109.5
H(43B)-C(43)-H(43C)	109.5
C(2)-C(44)-H(44A)	109.5
C(2)-C(44)-H(44B)	109.5
H(44A)-C(44)-H(44B)	109.5
C(2)-C(44)-H(44C)	109.5
H(44A)-C(44)-H(44C)	109.5
H(44B)-C(44)-H(44C)	109.5
O(113)-K(2)-O(105)	106.47(10)
O(113)-K(2)-O(101)	77.93(11)
O(105)-K(2)-O(101)	78.81(10)
O(113)-K(2)-O(111)	99.82(11)
O(105)-K(2)-O(111)	121.53(10)
O(101)-K(2)-O(111)	158.75(11)

O(113)-K(2)-O(112)	58.47(10)
O(105)-K(2)-O(112)	92.87(9)
O(101)-K(2)-O(112)	131.33(11)
O(111)-K(2)-O(112)	59.07(10)
O(113)-K(2)-O(108)	170.12(11)
O(105)-K(2)-O(108)	73.52(9)
O(101)-K(2)-O(108)	111.48(11)
O(111)-K(2)-O(108)	72.55(10)
O(112)-K(2)-O(108)	111.69(10)
O(113)-K(2)-O(109)	117.86(12)
O(105)-K(2)-O(109)	134.51(12)
O(101)-K(2)-O(109)	100.07(12)
O(111)-K(2)-O(109)	61.94(12)
O(112)-K(2)-O(109)	118.06(12)
O(108)-K(2)-O(109)	64.62(10)
O(113)-K(2)-O(102)	119.30(10)
O(105)-K(2)-O(102)	54.69(8)
O(101)-K(2)-O(102)	44.16(9)
O(111)-K(2)-O(102)	140.52(10)
O(112)-K(2)-O(102)	146.82(8)
O(108)-K(2)-O(102)	69.09(9)
O(109)-K(2)-O(102)	92.83(11)
O(113)-K(2)-H(105)	111(3)
O(105)-K(2)-H(105)	16.7(13)
O(101)-K(2)-H(105)	66(2)
O(111)-K(2)-H(105)	133(3)
O(112)-K(2)-H(105)	109.1(15)

O(108)-K(2)-H(105)	72(3)
O(109)-K(2)-H(105)	124(2)
O(102)-K(2)-H(105)	38.1(14)
O(113)-K(2)-H(108)	160(3)
O(105)-K(2)-H(108)	77(3)
O(101)-K(2)-H(108)	122(3)
O(111)-K(2)-H(108)	63(3)
O(112)-K(2)-H(108)	102(3)
O(108)-K(2)-H(108)	11(3)
O(109)-K(2)-H(108)	65(3)
O(102)-K(2)-H(108)	79(3)
H(105)-K(2)-H(108)	78(4)
O(113)-K(2)-H(113)	15.1(17)
O(105)-K(2)-H(113)	103(3)
O(101)-K(2)-H(113)	62.8(17)
O(111)-K(2)-H(113)	113.4(19)
O(112)-K(2)-H(113)	72.9(19)
O(108)-K(2)-H(113)	174.0(19)
O(109)-K(2)-H(113)	117(3)
O(102)-K(2)-H(113)	104.9(18)
H(105)-K(2)-H(113)	103(4)
H(108)-K(2)-H(113)	175(3)
C(101)-O(101)-K(2)	101.6(3)
C(101)-O(102)-K(2)	91.1(3)
C(103)-O(103)-C(107)	111.2(4)
C(113)-O(104)-C(109)	116.3(3)
C(113)-O(105)-K(2)	164.7(2)

C(113)-O(105)-H(105)	110(10)
K(2)-O(105)-H(105)	84(9)
C(119)-O(106)-C(115)	115.4(3)
C(119)-O(107)-H(107)	109.5
C(121)-O(108)-K(2)	107.1(3)
C(121)-O(108)-H(108)	131(10)
K(2)-O(108)-H(108)	32(10)
C(123)-O(109)-K(2)	104.2(3)
C(124)-O(110)-H(110)	109.5
C(128)-O(111)-C(125)	110.8(4)
C(128)-O(111)-K(2)	119.0(3)
C(125)-O(111)-K(2)	127.6(3)
C(129)-O(112)-C(133)	115.2(4)
C(129)-O(112)-K(2)	107.6(3)
C(133)-O(112)-K(2)	108.2(3)
C(132)-O(113)-K(2)	127.1(3)
C(132)-O(113)-H(113)	125(10)
K(2)-O(113)-H(113)	105(10)
O(101)-C(101)-O(102)	122.9(4)
O(101)-C(101)-C(102)	120.4(4)
O(102)-C(101)-C(102)	116.7(4)
C(103)-C(102)-C(101)	109.4(3)
C(103)-C(102)-C(144)	113.5(4)
C(101)-C(102)-C(144)	113.3(4)
C(103)-C(102)-H(102)	106.7
C(101)-C(102)-H(102)	106.7
C(144)-C(102)-H(102)	106.7

O(103)-C(103)-C(104)	110.8(4)
O(103)-C(103)-C(102)	105.7(4)
C(104)-C(103)-C(102)	116.3(4)
O(103)-C(103)-H(103)	107.9
C(104)-C(103)-H(103)	107.9
C(102)-C(103)-H(103)	107.9
C(103)-C(104)-C(143)	111.5(5)
C(103)-C(104)-C(105)	109.4(4)
C(143)-C(104)-C(105)	111.0(5)
C(103)-C(104)-H(104)	108.3
C(143)-C(104)-H(104)	108.3
C(105)-C(104)-H(104)	108.3
C(106)-C(105)-C(104)	111.4(5)
C(106)-C(105)-H(10B)	109.3
C(104)-C(105)-H(10B)	109.3
C(106)-C(105)-H(10C)	109.3
C(104)-C(105)-H(10C)	109.3
H(10B)-C(105)-H(10C)	108.0
C(105)-C(106)-C(107)	110.5(4)
C(105)-C(106)-H(10D)	109.6
C(107)-C(106)-H(10D)	109.6
C(105)-C(106)-H(10E)	109.6
C(107)-C(106)-H(10E)	109.6
H(10D)-C(106)-H(10E)	108.1
O(103)-C(107)-C(108)	107.7(4)
O(103)-C(107)-C(106)	109.0(4)
C(108)-C(107)-C(106)	116.0(4)

O(103)-C(107)-H(10F)	108.0
C(108)-C(107)-H(10F)	108.0
C(106)-C(107)-H(10F)	108.0
C(109)-C(108)-C(107)	113.0(3)
C(109)-C(108)-H(10G)	109.0
C(107)-C(108)-H(10G)	109.0
C(109)-C(108)-H(10H)	109.0
C(107)-C(108)-H(10H)	109.0
H(10G)-C(108)-H(10H)	107.8
O(104)-C(109)-C(110)	108.3(4)
O(104)-C(109)-C(108)	104.6(4)
C(110)-C(109)-C(108)	116.9(4)
O(104)-C(109)-H(109)	108.9
C(110)-C(109)-H(109)	108.9
C(108)-C(109)-H(109)	108.9
C(109)-C(110)-C(111)	107.5(4)
C(109)-C(110)-C(142)	113.0(5)
C(111)-C(110)-C(142)	111.5(5)
C(109)-C(110)-H(11C)	108.3
C(111)-C(110)-H(11C)	108.3
C(142)-C(110)-H(11C)	108.3
C(112)-C(111)-C(110)	113.8(4)
C(112)-C(111)-H(11D)	108.8
C(110)-C(111)-H(11D)	108.8
C(112)-C(111)-H(11E)	108.8
C(110)-C(111)-H(11E)	108.8
H(11D)-C(111)-H(11E)	107.7

C(111)-C(112)-C(113)	111.6(4)
C(111)-C(112)-C(141)	111.3(4)
C(113)-C(112)-C(141)	112.7(4)
C(111)-C(112)-H(112)	106.9
C(113)-C(112)-H(112)	106.9
C(141)-C(112)-H(112)	106.9
O(104)-C(113)-O(105)	110.4(3)
O(104)-C(113)-C(112)	111.1(3)
O(105)-C(113)-C(112)	106.7(3)
O(104)-C(113)-C(114)	103.1(3)
O(105)-C(113)-C(114)	111.3(3)
C(112)-C(113)-C(114)	114.1(4)
C(115)-C(114)-C(113)	115.4(4)
C(115)-C(114)-H(11F)	108.4
C(113)-C(114)-H(11F)	108.4
C(115)-C(114)-H(11G)	108.4
C(113)-C(114)-H(11G)	108.4
H(11F)-C(114)-H(11G)	107.5
O(106)-C(115)-C(116)	109.1(3)
O(106)-C(115)-C(114)	105.2(3)
C(116)-C(115)-C(114)	115.8(3)
O(106)-C(115)-H(115)	108.9
C(116)-C(115)-H(115)	108.9
C(114)-C(115)-H(115)	108.9
C(115)-C(116)-C(117)	110.8(4)
C(115)-C(116)-C(140)	111.0(4)
C(117)-C(116)-C(140)	109.2(4)

C(115)-C(116)-H(116)	108.6
C(117)-C(116)-H(116)	108.6
C(140)-C(116)-H(116)	108.6
C(116)-C(117)-C(118)	113.3(4)
C(116)-C(117)-H(11H)	108.9
C(118)-C(117)-H(11H)	108.9
C(116)-C(117)-H(11I)	108.9
C(118)-C(117)-H(11I)	108.9
H(11H)-C(117)-H(11I)	107.7
C(139)-C(118)-C(117)	111.1(4)
C(139)-C(118)-C(119)	111.9(4)
C(117)-C(118)-C(119)	108.0(4)
C(139)-C(118)-H(118)	108.6
C(117)-C(118)-H(118)	108.6
C(119)-C(118)-H(118)	108.6
O(107)-C(119)-O(106)	110.8(3)
O(107)-C(119)-C(118)	107.9(4)
O(106)-C(119)-C(118)	110.1(3)
O(107)-C(119)-C(120)	111.1(4)
O(106)-C(119)-C(120)	103.3(3)
C(118)-C(119)-C(120)	113.7(4)
C(121)-C(120)-C(119)	113.6(4)
C(121)-C(120)-H(12A)	108.8
C(119)-C(120)-H(12A)	108.8
C(121)-C(120)-H(12B)	108.8
C(119)-C(120)-H(12B)	108.8
H(12A)-C(120)-H(12B)	107.7

O(108)-C(121)-C(120)	112.1(3)
O(108)-C(121)-C(122)	106.6(4)
C(120)-C(121)-C(122)	112.2(4)
O(108)-C(121)-H(121)	108.6
C(120)-C(121)-H(121)	108.6
C(122)-C(121)-H(121)	108.6
C(123)-C(122)-C(138)	110.6(5)
C(123)-C(122)-C(121)	106.7(4)
C(138)-C(122)-C(121)	110.5(4)
C(123)-C(122)-H(122)	109.7
C(138)-C(122)-H(122)	109.7
C(121)-C(122)-H(122)	109.7
O(109)-C(123)-C(124)	120.7(6)
O(109)-C(123)-C(122)	120.1(5)
C(124)-C(123)-C(122)	119.2(5)
O(110)-C(124)-C(125)	111.1(5)
O(110)-C(124)-C(123)	110.3(4)
C(125)-C(124)-C(123)	112.9(5)
O(110)-C(124)-C(137)	105.3(4)
C(125)-C(124)-C(137)	110.1(5)
C(123)-C(124)-C(137)	106.9(5)
O(111)-C(125)-C(124)	108.2(4)
O(111)-C(125)-C(126)	104.1(5)
C(124)-C(125)-C(126)	116.3(5)
O(111)-C(125)-H(125)	109.3
C(124)-C(125)-H(125)	109.3
C(126)-C(125)-H(125)	109.3

C(127)-C(126)-C(125)	102.6(5)
C(127)-C(126)-H(12C)	111.2
C(125)-C(126)-H(12C)	111.2
C(127)-C(126)-H(12D)	111.2
C(125)-C(126)-H(12D)	111.2
H(12C)-C(126)-H(12D)	109.2
C(126)-C(127)-C(128)	101.9(5)
C(126)-C(127)-H(12E)	111.4
C(128)-C(127)-H(12E)	111.4
C(126)-C(127)-H(12F)	111.4
C(128)-C(127)-H(12F)	111.4
H(12E)-C(127)-H(12F)	109.2
O(111)-C(128)-C(136)	108.8(5)
O(111)-C(128)-C(129)	109.0(4)
C(136)-C(128)-C(129)	109.7(5)
O(111)-C(128)-C(127)	105.9(5)
C(136)-C(128)-C(127)	111.2(6)
C(129)-C(128)-C(127)	112.2(5)
O(112)-C(129)-C(130)	110.1(4)
O(112)-C(129)-C(128)	106.1(4)
C(130)-C(129)-C(128)	115.9(5)
O(112)-C(129)-H(129)	108.2
C(130)-C(129)-H(129)	108.2
C(128)-C(129)-H(129)	108.2
C(131)-C(130)-C(129)	111.5(5)
C(131)-C(130)-H(13A)	109.3
C(129)-C(130)-H(13A)	109.3

C(131)-C(130)-H(13B)	109.3
C(129)-C(130)-H(13B)	109.3
H(13A)-C(130)-H(13B)	108.0
C(130)-C(131)-C(132)	112.0(4)
C(130)-C(131)-H(13C)	109.2
C(132)-C(131)-H(13C)	109.2
C(130)-C(131)-H(13D)	109.2
C(132)-C(131)-H(13D)	109.2
H(13C)-C(131)-H(13D)	107.9
O(113)-C(132)-C(135)	111.1(4)
O(113)-C(132)-C(133)	106.3(4)
C(135)-C(132)-C(133)	113.0(4)
O(113)-C(132)-C(131)	106.1(4)
C(135)-C(132)-C(131)	111.8(4)
C(133)-C(132)-C(131)	108.1(4)
O(112)-C(133)-C(134)	111.5(5)
O(112)-C(133)-C(132)	109.2(4)
C(134)-C(133)-C(132)	115.6(5)
O(112)-C(133)-H(133)	106.7
C(134)-C(133)-H(133)	106.7
C(132)-C(133)-H(133)	106.7
C(133)-C(134)-H(13E)	109.5
C(133)-C(134)-H(13F)	109.5
H(13E)-C(134)-H(13F)	109.5
C(133)-C(134)-H(13G)	109.5
H(13E)-C(134)-H(13G)	109.5
H(13F)-C(134)-H(13G)	109.5

C(132)-C(135)-H(13H)	109.5
C(132)-C(135)-H(13I)	109.5
H(13H)-C(135)-H(13I)	109.5
C(132)-C(135)-H(13J)	109.5
H(13H)-C(135)-H(13J)	109.5
H(13I)-C(135)-H(13J)	109.5
C(128)-C(136)-H(13K)	109.5
C(128)-C(136)-H(13L)	109.5
H(13K)-C(136)-H(13L)	109.5
C(128)-C(136)-H(13M)	109.5
H(13K)-C(136)-H(13M)	109.5
H(13L)-C(136)-H(13M)	109.5
C(124)-C(137)-H(13N)	109.5
C(124)-C(137)-H(13O)	109.5
H(13N)-C(137)-H(13O)	109.5
C(124)-C(137)-H(13P)	109.5
H(13N)-C(137)-H(13P)	109.5
H(13O)-C(137)-H(13P)	109.5
C(122)-C(138)-H(13Q)	109.5
C(122)-C(138)-H(13R)	109.5
H(13Q)-C(138)-H(13R)	109.5
C(122)-C(138)-H(13S)	109.5
H(13Q)-C(138)-H(13S)	109.5
H(13R)-C(138)-H(13S)	109.5
C(118)-C(139)-H(13T)	109.5
C(118)-C(139)-H(13U)	109.5
H(13T)-C(139)-H(13U)	109.5

C(118)-C(139)-H(13V)	109.5
H(13T)-C(139)-H(13V)	109.5
H(13U)-C(139)-H(13V)	109.5
C(116)-C(140)-H(14C)	109.5
C(116)-C(140)-H(14D)	109.5
H(14C)-C(140)-H(14D)	109.5
C(116)-C(140)-H(14E)	109.5
H(14C)-C(140)-H(14E)	109.5
H(14D)-C(140)-H(14E)	109.5
C(112)-C(141)-H(14F)	109.5
C(112)-C(141)-H(14G)	109.5
H(14F)-C(141)-H(14G)	109.5
C(112)-C(141)-H(14H)	109.5
H(14F)-C(141)-H(14H)	109.5
H(14G)-C(141)-H(14H)	109.5
C(110)-C(142)-H(14I)	109.5
C(110)-C(142)-H(14J)	109.5
H(14I)-C(142)-H(14J)	109.5
C(110)-C(142)-H(14K)	109.5
H(14I)-C(142)-H(14K)	109.5
H(14J)-C(142)-H(14K)	109.5
C(104)-C(143)-H(14L)	109.5
C(104)-C(143)-H(14M)	109.5
H(14L)-C(143)-H(14M)	109.5
C(104)-C(143)-H(14N)	109.5
H(14L)-C(143)-H(14N)	109.5
H(14M)-C(143)-H(14N)	109.5

C(102)-C(144)-H(14O)	109.5
C(102)-C(144)-H(14P)	109.5
H(14O)-C(144)-H(14P)	109.5
C(102)-C(144)-H(14Q)	109.5
H(14O)-C(144)-H(14Q)	109.5
H(14P)-C(144)-H(14Q)	109.5
H(201)-O(201)-H(202)	152(10)
O(202)-C(201)-C(202)	122.2(9)
O(202)-C(201)-C(203)	121.7(13)
C(202)-C(201)-C(203)	115.7(11)
O(302)-C(301)-C(302)	117(3)
O(302)-C(301)-C(303)	125(3)
C(302)-C(301)-C(303)	118(2)
C(301)-C(302)-H(30C)	109.5
C(301)-C(302)-H(30D)	109.5
H(30C)-C(302)-H(30D)	109.5
C(301)-C(302)-H(30E)	109.5
H(30C)-C(302)-H(30E)	109.5
H(30D)-C(302)-H(30E)	109.5
C(301)-C(303)-H(30F)	109.5
C(301)-C(303)-H(30G)	109.5
H(30F)-C(303)-H(30G)	109.5
C(301)-C(303)-H(30H)	109.5
H(30F)-C(303)-H(30H)	109.5
H(30G)-C(303)-H(30H)	109.5

Table S5. Anisotropic displacement parameters ($Å^2 x 10^3$) for terrosamycin A (**1**). The anisotropic displacement factor exponent take the form: $-2\pi^2[h^2 a^{*2}U^{11} + ... + 2 h k a^* b^* U^{12}]$.

	U^{11}	U^{22}	U^{33}	U^{23}	U^{13}	U^{12}
K(1)	32(1)	40(1)	35(1)	1(1)	2(1)	-4(1)
O(1)	40(2)	30(2)	36(1)	1(1)	12(1)	-1(1)
O(2)	54(2)	32(2)	40(2)	4(1)	1(2)	-2(2)
O(3)	33(2)	36(2)	35(1)	3(1)	3(1)	-2(1)
O(4)	30(2)	40(2)	34(1)	10(1)	6(1)	0(1)
O(5)	32(2)	46(2)	30(1)	-4(1)	5(1)	9(2)
O(6)	35(2)	33(2)	30(1)	-1(1)	11(1)	0(1)
O(7)	52(2)	30(2)	29(1)	0(1)	13(1)	-2(2)
O(8)	32(2)	53(2)	37(1)	-1(1)	12(1)	-8(2)
O(9)	54(2)	47(2)	58(2)	-4(2)	12(2)	-2(2)
O(10)	51(2)	42(2)	60(2)	-5(2)	10(2)	-1(2)
O(11)	34(2)	56(2)	42(2)	-9(2)	2(1)	-13(2)
O(12)	44(2)	36(2)	44(2)	-1(1)	9(1)	7(2)
O(13)	78(3)	40(2)	30(1)	0(1)	3(2)	-11(2)
C(1)	38(2)	33(2)	34(2)	1(2)	18(2)	-2(2)
C(2)	47(3)	39(3)	31(2)	-5(2)	15(2)	-8(2)
C(3)	59(3)	33(2)	28(2)	-3(2)	3(2)	-16(2)
C(4)	77(4)	45(3)	36(2)	-8(2)	-3(2)	-15(3)
C(5)	77(5)	54(3)	54(3)	-9(3)	-19(3)	-15(3)
C(6)	34(3)	61(4)	76(3)	-1(3)	-9(3)	-1(3)
C(7)	34(3)	42(3)	49(2)	1(2)	-1(2)	-7(2)
C(8)	32(3)	41(3)	73(3)	9(2)	14(2)	4(2)

C(9)	31(2)	38(2)	41(2)	12(2)	14(2)	7(2)
C(10)	70(4)	53(3)	50(3)	18(2)	29(3)	11(3)
C(11)	71(4)	70(4)	35(2)	18(2)	23(2)	26(3)
C(12)	54(3)	68(3)	23(2)	10(2)	12(2)	24(3)
C(13)	25(2)	49(3)	25(2)	4(2)	8(2)	4(2)
C(14)	35(2)	36(2)	28(2)	-4(2)	10(2)	0(2)
C(15)	40(3)	29(2)	26(2)	0(2)	8(2)	7(2)
C(16)	45(3)	49(3)	37(2)	1(2)	8(2)	11(2)
C(17)	58(3)	42(3)	30(2)	5(2)	2(2)	19(2)
C(18)	58(3)	42(3)	24(2)	4(2)	14(2)	5(2)
C(19)	50(3)	30(2)	28(2)	6(2)	15(2)	6(2)
C(20)	50(3)	41(3)	36(2)	2(2)	23(2)	-5(2)
C(21)	33(2)	36(2)	40(2)	-1(2)	18(2)	-4(2)
C(22)	35(3)	54(3)	46(2)	0(2)	16(2)	-11(2)
C(23)	26(2)	43(3)	56(2)	-2(2)	16(2)	-6(2)
C(24)	28(2)	47(3)	58(3)	-4(2)	8(2)	-4(2)
C(25)	24(2)	51(3)	58(3)	-3(2)	0(2)	-6(2)
C(26)	44(3)	76(4)	72(3)	-4(3)	-1(3)	-27(3)
C(27)	58(4)	86(5)	51(3)	-9(3)	-11(3)	-28(4)
C(28)	49(3)	48(3)	44(2)	-6(2)	7(2)	-9(3)
C(29)	34(3)	52(3)	33(2)	-9(2)	2(2)	-4(2)
C(30)	28(3)	60(3)	52(3)	4(2)	-8(2)	4(2)
C(31)	61(4)	54(3)	44(2)	0(2)	6(2)	12(3)
C(32)	66(4)	40(3)	27(2)	1(2)	7(2)	0(3)
C(33)	42(3)	46(3)	43(2)	0(2)	5(2)	0(2)
C(34)	94(5)	63(4)	59(3)	-1(3)	37(3)	1(4)
C(35)	84(4)	42(3)	43(2)	6(2)	9(3)	-3(3)

C(36)	82(5)	46(3)	63(3)	-14(3)	22(3)	-14(3)
C(37)	34(3)	61(4)	74(3)	7(3)	7(3)	-10(3)
C(38)	47(3)	93(5)	60(3)	-15(3)	26(3)	-12(3)
C(39)	76(4)	65(4)	33(2)	16(2)	23(2)	9(3)
C(40)	54(4)	82(4)	50(3)	13(3)	12(3)	32(3)
C(41)	75(4)	71(4)	33(2)	-14(2)	6(2)	14(3)
C(42)	130(7)	70(4)	68(3)	26(3)	51(4)	-2(4)
C(43)	115(6)	87(5)	30(2)	-8(3)	4(3)	-27(4)
C(44)	52(3)	38(3)	51(2)	-8(2)	13(2)	-2(2)
K(2)	44(1)	40(1)	32(1)	2(1)	2(1)	0(1)
O(101)	53(2)	33(2)	55(2)	1(2)	1(2)	-5(2)
O(102)	38(2)	30(2)	39(1)	2(1)	9(1)	4(1)
O(103)	38(2)	39(2)	31(1)	0(1)	2(1)	-2(2)
O(104)	36(2)	34(2)	26(1)	1(1)	7(1)	-7(1)
O(105)	35(2)	40(2)	21(1)	-3(1)	2(1)	6(1)
O(106)	29(2)	33(2)	30(1)	4(1)	8(1)	0(1)
O(107)	32(2)	39(2)	49(2)	3(1)	17(1)	7(1)
O(108)	43(2)	60(2)	26(1)	3(1)	12(1)	2(2)
O(109)	110(4)	40(2)	57(2)	-4(2)	25(2)	3(2)
O(110)	66(3)	36(2)	45(2)	-1(1)	12(2)	3(2)
O(111)	53(2)	63(2)	37(2)	14(2)	-3(2)	-1(2)
O(112)	48(2)	33(2)	47(2)	-4(1)	1(2)	1(2)
O(113)	43(2)	37(2)	64(2)	3(2)	8(2)	3(2)
C(101)	44(3)	38(3)	27(2)	3(2)	16(2)	-4(2)
C(102)	36(3)	33(2)	49(2)	6(2)	19(2)	4(2)
C(103)	27(2)	40(3)	52(2)	10(2)	1(2)	-3(2)
C(104)	30(3)	49(3)	72(3)	10(3)	3(2)	8(2)

C(105)	52(4)	59(4)	78(4)	15(3)	-27(3)	8(3)
C(106)	65(4)	53(3)	42(2)	7(2)	-12(2)	5(3)
C(107)	56(3)	38(3)	30(2)	2(2)	-5(2)	-1(2)
C(108)	58(3)	36(2)	28(2)	4(2)	6(2)	-9(2)
C(109)	44(3)	36(2)	28(2)	-2(2)	11(2)	-13(2)
C(110)	60(3)	44(3)	50(2)	-3(2)	30(2)	-10(3)
C(111)	38(3)	51(3)	65(3)	-9(2)	30(2)	-3(3)
C(112)	33(2)	44(3)	52(2)	-16(2)	19(2)	-9(2)
C(113)	30(2)	35(2)	27(2)	-5(2)	6(2)	3(2)
C(114)	36(2)	34(2)	26(2)	3(2)	12(2)	0(2)
C(115)	29(2)	38(2)	26(2)	6(2)	9(2)	6(2)
C(116)	38(3)	34(2)	36(2)	3(2)	2(2)	0(2)
C(117)	34(3)	41(3)	52(2)	-3(2)	0(2)	-4(2)
C(118)	35(3)	30(2)	64(3)	-1(2)	15(2)	-6(2)
C(119)	28(2)	32(2)	42(2)	6(2)	15(2)	-4(2)
C(120)	45(3)	44(3)	40(2)	4(2)	19(2)	4(2)
C(121)	56(3)	43(3)	30(2)	7(2)	23(2)	11(2)
C(122)	60(3)	50(3)	32(2)	10(2)	22(2)	7(3)
C(123)	81(4)	47(3)	24(2)	0(2)	20(2)	1(3)
C(124)	86(4)	49(3)	23(2)	3(2)	12(2)	-7(3)
C(125)	76(4)	61(4)	34(2)	10(2)	-3(2)	1(3)
C(126)	87(5)	75(4)	73(4)	37(4)	-28(4)	-16(4)
C(127)	75(5)	100(6)	73(4)	36(4)	-23(4)	0(4)
C(128)	50(3)	42(3)	55(3)	8(2)	-13(2)	-5(3)
C(129)	40(3)	44(3)	64(3)	3(2)	-8(2)	-4(2)
C(130)	67(4)	56(3)	42(2)	-9(2)	-10(2)	-7(3)
C(131)	50(3)	53(3)	61(3)	-13(3)	-11(3)	-7(3)

C(132)	43(3)	33(2)	53(2)	1(2)	-1(2)	-5(2)
C(133)	42(3)	39(3)	55(3)	-7(2)	9(2)	0(2)
C(134)	66(4)	63(4)	113(5)	-3(4)	38(4)	1(4)
C(135)	49(3)	47(3)	92(4)	-3(3)	13(3)	-3(3)
C(136)	68(4)	34(3)	121(5)	-2(3)	9(4)	5(3)
C(137)	136(7)	65(4)	32(2)	11(2)	22(3)	-5(4)
C(138)	89(5)	89(5)	42(2)	8(3)	36(3)	27(4)
C(139)	40(3)	59(4)	90(4)	-9(3)	27(3)	-20(3)
C(140)	51(3)	72(4)	34(2)	6(2)	-2(2)	-6(3)
C(141)	36(3)	56(3)	73(3)	-5(3)	7(3)	11(3)
C(142)	77(5)	64(4)	86(4)	1(3)	45(4)	-23(4)
C(143)	29(3)	90(5)	118(5)	11(4)	7(3)	18(3)
C(144)	65(4)	40(3)	59(3)	4(2)	23(3)	13(3)
O(201)	52(2)	33(2)	46(2)	-3(1)	6(2)	-2(2)
O(202)	188(10)	116(6)	62(4)	10(4)	7(4)	17(6)
C(201)	88(7)	86(7)	42(4)	-8(4)	25(4)	-6(6)
C(202)	97(7)	67(7)	77(7)	-19(5)	34(6)	-22(6)
C(203)	260(20)	90(13)	59(5)	-6(14)	28(12)	46(16)
O(302)	188(10)	116(6)	62(4)	10(4)	7(4)	17(6)
C(301)	88(7)	86(7)	42(4)	-8(4)	25(4)	-6(6)
C(302)	97(7)	67(7)	77(7)	-19(5)	34(6)	-22(6)
C(303)	260(20)	90(13)	59(5)	-6(14)	28(12)	46(16)

Table S6. Hydrogen coordinates (x10^4) and isotropic displacement parameters (Å^{2}x10^3) for terrosamycin A (**1**).

	x	y	z	U(eq)
H(7)	9552	7482	12058	54
H(10)	5690	6511	9126	78
H(2)	9290	8317	12453	46
H(3)	10892	7982	12404	50
H(4)	11698	8727	13022	66
H(5A)	12908	8046	13724	81
H(5B)	13406	8459	14022	81
H(6A)	13445	8568	12109	73
H(6B)	14086	8178	12479	73
H(7A)	12398	7847	11633	53
H(8A)	12875	8352	9970	58
H(8B)	13523	7963	10320	58
H(9)	11128	8029	9268	43
H(10A)	12852	7711	8248	66
H(11A)	11349	7586	6673	68
H(11B)	10453	7776	7290	68
H(12)	11642	7087	7947	57
H(14A)	11808	6834	9862	39
H(14B)	10462	6754	9463	39
H(15)	11166	7231	11389	38
H(16)	11500	6440	11637	53

H(17A)	11805	6545	13634	54
H(17B)	11624	6985	13433	54
H(18)	9891	6432	12747	49
H(20A)	7855	6920	12542	48
H(20B)	8042	6621	11618	48
H(21)	7623	7394	11067	42
H(22)	5974	6797	10734	53
H(25)	4296	7401	7528	56
H(26A)	4297	6674	6533	80
H(26B)	3249	6968	6346	80
H(27A)	4029	7343	5186	82
H(27B)	4398	6936	4820	82
H(29)	6079	7519	4644	49
H(30A)	4745	7906	5188	60
H(30B)	5557	7956	6446	60
H(31A)	5759	8487	5423	65
H(31B)	5983	8245	4376	65
H(33)	8738	7901	6042	53
H(34A)	8278	7555	4294	104
H(34B)	8599	7983	4143	104
H(34C)	7286	7853	3809	104
H(35A)	7687	8830	5620	86
H(35B)	7853	8598	4533	86
H(35C)	8823	8586	5706	86
H(36A)	7299	6941	6319	94
H(36B)	6491	6826	5106	94
H(36C)	6247	6661	6263	94

H(37A)	3542	6646	8343	86
H(37B)	3396	7041	8917	86
H(37C)	4110	6713	9674	86
H(38A)	6029	7138	12429	96
H(38B)	4868	7249	11522	96
H(38C)	5944	7526	11751	96
H(39A)	10277	6539	14734	85
H(39B)	9039	6707	14154	85
H(39C)	10120	6980	14534	85
H(40A)	13159	6704	11341	93
H(40B)	13388	6597	12669	93
H(40C)	13145	7022	12275	93
H(41A)	9244	7204	7166	91
H(41B)	9834	6809	7557	91
H(41C)	10044	7012	6438	91
H(42A)	12372	8199	6840	127
H(42B)	12943	8364	8082	127
H(42C)	11584	8389	7582	127
H(43A)	11209	8153	14516	120
H(43B)	10418	8512	14135	120
H(43C)	11685	8560	14928	120
H(44A)	9810	8931	12370	70
H(44B)	8732	8861	11314	70
H(44C)	9998	8881	11105	70
H(107)	5903	5314	2173	58
H(110)	1067	6145	-1036	74
H(102)	5467	4404	2236	45

H(103)	5439	4731	3884	49
H(104)	6138	3986	4583	62
H(10B)	7175	4246	6306	84
H(10C)	6849	4662	5852	84
H(10D)	5663	4510	7057	69
H(10E)	5296	4124	6400	69
H(10F)	4725	4848	5405	52
H(10G)	3147	4322	5943	50
H(10H)	3490	4709	6599	50
H(109)	2285	4642	4248	43
H(11C)	1437	4970	6056	58
H(11D)	311	4893	3678	58
H(11E)	-228	5094	4616	58
H(112)	1039	5580	4804	50
H(11F)	2491	5906	3478	38
H(11G)	2883	5850	4833	38
H(115)	4364	5429	4159	37
H(116)	4687	6208	4687	44
H(11H)	6465	5674	4690	53
H(11I)	6658	6109	4982	53
H(118)	5823	6272	3076	51
H(12A)	4670	6109	1114	50
H(12B)	5576	5805	911	50
H(121)	4081	5341	703	49
H(122)	3836	5933	-970	55
H(125)	489	5389	-2655	72
H(12C)	-364	6129	-2443	104

H(12D)	-633	5867	-3567	104
H(12E)	-1823	5480	-2827	107
H(12F)	-2122	5882	-2360	107
H(129)	-2315	5266	-811	63
H(13A)	-1839	4906	-2242	71
H(13B)	-577	4831	-1465	71
H(13C)	-1668	4305	-1374	70
H(13D)	-2659	4550	-1047	70
H(133)	-908	4821	1767	55
H(13E)	-2651	5184	1295	117
H(13F)	-2777	4759	1659	117
H(13G)	-3134	4876	336	117
H(13H)	-1496	3912	474	95
H(13I)	-2524	4157	725	95
H(13J)	-1338	4125	1673	95
H(13K)	-570	5811	527	115
H(13L)	-1791	5937	-241	115
H(13M)	-639	6114	-464	115
H(13N)	1331	6108	-3390	116
H(13O)	1990	5728	-3518	116
H(13P)	2674	6071	-2816	116
H(13Q)	4772	5193	-874	105
H(13R)	5453	5575	-925	105
H(13S)	4520	5434	-2030	105
H(13T)	7513	5706	3159	92
H(13U)	7788	6136	3463	92
H(13V)	7186	6016	2177	92

H(14C)	4357	5837	6251	81
H(14D)	5624	6009	6561	81
H(14E)	5431	5580	6190	81
H(14F)	567	5839	2915	84
H(14G)	-528	5671	3281	84
H(14H)	59	5443	2428	84
H(14I)	638	4292	4846	107
H(14J)	19	4491	5729	107
H(14K)	1272	4315	6177	107
H(14L)	7231	4177	3345	121
H(14M)	8040	4218	4606	121
H(14N)	7449	4580	3933	121
H(14O)	5412	3782	2790	80
H(14P)	4403	3849	1669	80
H(14Q)	4116	3826	2901	80
H(20C)	6530	8486	142	117
H(20D)	5248	8424	277	117
H(20E)	6293	8209	1104	117
H(20F)	5233	9161	561	208
H(20G)	6546	9170	486	208
H(20H)	6198	9322	1608	208
H(30C)	5589	8460	1570	117
H(30D)	6836	8616	1561	117
H(30E)	6021	8454	409	117
H(30F)	5920	9520	780	208
H(30G)	6901	9255	517	208
H(30H)	6767	9301	1803	208

H(5)	9480(110)	7630(30)	8700(100)	208
H(8)	8360(50)	7030(40)	10070(110)	208
H(13)	7720(130)	8460(30)	7420(100)	208
H(105)	2730(80)	5100(40)	2870(110)	208
H(108)	2210(70)	5600(40)	560(110)	208
H(113)	350(110)	4260(30)	640(110)	208
H(201)	8050(90)	9010(40)	8330(110)	208
H(202)	9450(70)	8840(50)	8560(120)	208

Table S7. Hydrogen bonds for terrosamycin A (**1**) [Å and °].

D-H...A	d(D-H)	d(H...A)	d(D...A)	<(DHA)
O(7)-H(7)...O(1)	0.84	1.82	2.660(4)	175.6
C(21)-H(21)...O(1)	1.00	2.38	3.165(5)	134.9
C(35)-H(35B)...O(202)	0.98	2.60	3.490(9)	151.9
C(121)-H(121)...O(102)	1.00	2.38	3.192(5)	138.0
C(135)-H(13I)...O(302)#1	0.98	2.61	3.48(2)	148.0
C(202)-H(20C)...O(2)#2	0.98	2.60	3.400(13)	138.9
C(303)-H(30G)...O(101)#3	0.98	2.57	3.37(7)	139.8
O(8)-H(8)...O(6)	0.81(6)	1.86(7)	2.641(4)	161(14)
O(13)-H(13)...O(201)	0.81(6)	2.04(8)	2.811(5)	160(15)
O(105)-H(105)...O(102)	0.81(6)	1.91(8)	2.664(4)	155(13)
O(113)-H(113)...O(201)#4	0.81(6)	2.01(7)	2.797(5)	164(14)
O(201)-H(201)...O(101)#5	0.90(9)	1.85(10)	2.712(5)	161(14)

Symmetry transformations used to generate equivalent atoms:

#1 -x,y-1/2,-z #2 x,y,z-1 #3 -x+1,y+1/2,-z

#4 -x+1,y-1/2,-z+1 #5 -x+1,y+1/2,-z+1
